# History of the German-language ENT journals

**DOI:** 10.1007/s00106-021-01036-x

**Published:** 2021-06-01

**Authors:** Norbert Stasche, Michael Bärmann

**Affiliations:** 1 Medizinische Fakultät Mannheim, Universität Heidelberg, Theodor-Kutzer-Ufer 1–3, 68167 Mannheim, Germany; 2 ENT Clinic, Westpfalz-Klinikum GmbH, Hellmut-Hartert Str. 1, 67655 Kaiserslautern, Germany

**Keywords:** Medical journalism, Journals as topic, Information dissemination, Serial publications, Scholarly Communication

## Abstract

In 1864, the worldwide oldest journal in an area of the later established specialty of otorhinolaryngology was founded as the German *Archiv für Ohrenheilkunde* (“Archive of Otology”) by its first editors Anton von Tröltsch (Würzburg), Adam Politzer (Vienna), and Hermann Schwartze (Halle/S.). Ear, nose, and throat (ENT) topics had previously been published in universal medical journals. In the next few decades, numerous journals in the field of ENT were founded, the eventful history of which is presented up to the present day. Particular attention is paid to the historical and personal context of the editors of newly founded magazines and their publishers. The journal landscape, which was changing through acquisitions and mergers of publishers, is described in detail. The merging of the specialties of otology and laryngo-rhinology in Germany, which lasted until the 1920s, had a profound influence on journal titles and contents. An attempt is made to present the most important titles in their historical development. All the important editors of the German ENT journals are mentioned, although it was not possible to include the names of the editors of the current journals, which are becoming more and more numerous. One chapter deals exclusively with the development of journal publishers. The inserted tables and figures will help to resolve some of the confusion caused by repeated similar names of journals by showing their historical development.

## Introduction

Johann Just von Berger (1723–1791) from Celle, the royal personal physician to Christian VII, King of Denmark and Norway since 1774, suffered from severe ear disease with dizzy spells, progressive deafness and agonizing noises in the last years of his life. Two hundred fifty years ago, treatment options for inner ear diseases were limited—with surgical intervention used as a last resort in desperate cases. The physician’s suffering was probably enormous. After trepanation of the mastoid and subsequent wound irrigation, the patient died in Copenhagen in 1791 of purulent meningitis [8]. It was the first known death after the opening of the mastoid. Because of the prominence of the patient at the Danish court, the case quickly became widely known, and mastoid surgery was spurned as malpractice until the middle of the 19th century. When Hermann Schwartze (1837–1910) first published a scientific basis for mastoidectomy, “Über die Eröffnung des Warzenfortsatzes” (Concerning the opening of the mastoid process) in 1873, the tragic case gained renewed attention when it was mentioned in the considerations. For many years, Baron Dr. von Berger was considered a martyr of ear surgery [83]. Schwartze, a pioneer in ear surgery from Halle, was co-editor and one of the founders of the *Archiv für Ohrenheilkunde*, the world’s oldest journal exclusively dedicated to one area of what would later become ear, nose and throat medicine. *European Archives of Oto-Rhino-Laryngology and Head & Neck* is still published today.

Up until the beginning of the 19th century, doctors exchanged information about illnesses, new therapies and medical experiences exclusively through letters, personal visits or textbooks. Only with the development of medicine as a natural science did it become necessary to find other forms of communication in order to keep pace with rapid developments. In 1670, the world’s first medical journal was launched as *Miscellanea curiosa medico-physica Academiae Naturae Curiosorum sive Ephemeridum medico-physicarum germanicarum curiosarum* by the members of the Leopoldina (Academia Naturae Curiosorum), founded in 1652. Rudolf Virchow (1821–1902) described the importance of this first foundation of a medical periodical in 1865 as follows: “Von seiner Gründung in den letzten Decennien des 17. Jahrhunderts an hat es als ein Vorbild gedient, zunächst für andere Gesellschaftsschriften, sodann, namentlich seit dem Ende des vorigen Jahrhunderts, für immer zahlreichere, theils universelle, theils specialistische Fachjournale” (From its foundation in the last decade of the 17th century it has served as a model, first for publications of other societies, then, specifically since then at the end of the last century, for increasingly numerous general and speciality journals) [100]. ENT topics were already represented in the first edition of the first volume of the *Miscellanea curiosa*. Philipp Jacob Sachs (1627–1672) wrote about possible connections between the ear and other anatomical regions in Latin, the language of the educated at that time [71]. Even 175 years later, in 1845, this text was quoted in the practical handbook of otology by Martell Frank (1810–1889) [30].

Today, 350 years later, the exchange of scientific information is largely digitized and internet-based. The scientific journals with their peer-review procedures still represent the “container”, that is, the external form of scientific exchange. In addition, scientific conventions, practical scientific courses and clinic visits are still widely practiced methods for the exchange of scientific information and the dissemination of new methods. This presentation intends to illuminate the historical development of ENT medicine in Germany from the perspective of German-language, scientific ENT journals.

## Founding phase and development in the 19th century

In addition to the *Miscellanea curiosa*, a number of other medical journals were founded at the end of the 18th century, which witnessed a broadening scientific basis for medicine. Christoph Wilhelm Hufeland (1762–1836), the personal physician of Friedrich Wilhelm III, King of Prussia, founded one of the most widespread journals at the time, the *Journal der praktischen Arzneikunde und Wundarzneikunst* in 1795, which covered the most diverse directions of medicine from homeopathy to animal magnetism [5]. The journal was published in 83 volumes up to Hufeland’s death and was one of the most respected, comprehensive and instructive medical journals in the German language. The successful publication of the journal also brought Hufeland some prosperity: “Außer dem wissenschaftlichen Nutzen, den das für die Aufrechterhaltung der erfahrungsmäßigen Medicin (im Gegensatz zur hypothetischen) bestimmte ‘Journal der praktischen Heilkunde’ stiftete, wurde es auch für H. eine gute Stütze in der Noth, eine Hauptquelle seines Vermögens, indem er sich zum Grundsatz machte, die Einkünfte davon nicht auszugeben, sondern zurückzulegen. … Durch seine litterarischen Arbeiten, besonders die Makrobiotik und das Journal hatte er so viel gewonnen, daß er ein Capital von 10,000 Thlrn. besaß, …” (In addition to the scientific usefulness of the ‘Journal of Practical Medicine’, which was intended for maintaining empirical medicine (as opposed to hypothetical), the journal was also a financial support for H. It helped build his fortune, as he made it a principle to save, rather than spend the income generated from it … Through his literary work, especially macrobiotics, and the journal, he had accrued capital of 10,000 thalers …) [34].

With the establishment of the German Empire in 1871 under Prussian leadership, a nation-state emerged in Germany with a uniform market and uniform rules. The liberal character of the emerging civil society promoted education and science, especially in the Reich capital Berlin. It is therefore not surprising that during this time essential impulses for medical developments came from Berlin and especially from the Charité. The revolution of 1848/49 had laid the foundation for democratic development in Germany, even if its failure initially set back the development of the political bourgeoisie with its free thinking and ideas of a free economy. The onset of industrialization and accelerated economic development in the last third of the 19th century led to rapid expansion of the transportation infrastructure. Countless new citizens immigrated to the imperial capital Berlin to work for the expanding industrial companies. This industrial revolution led to a concentration of the population in the big cities with the consequence of an unprecedented need for medical care within small geographical areas. The social legislation initiated by Reich Chancellor Bismarck at this time, with the introduction of mandatory health and accident insurance for workers (1883/84) and the introduction of pension insurance in 1891, led to an improvement in social wellbeing (cf. Draheim 2014 [26]).

An influx in the number of medical students and increasing prosperity among doctors in Germany quickly led to the establishment of medical publishing companies in the second half of the 19th century. In 1878 there were 425 medical titles and 67 journals from 150 publishing companies. Parallel to the increasing specialization in medicine, there was also a specialization in medical publishing. This culminated in the successful founding of the first exclusively medical publishers by Georg Thieme (1860–1925) in Leipzig in 1886 and Samuel Karger (1863–1935) in Berlin in 1890. Programs and individual journals sometimes changed publishing companies, leading to a specialization of some for medicine. Numerous publishing houses such as F. C. W. Vogel, Leipzig, J. F. Bergmann, Wiesbaden, Gustav Fischer, Jena, J. F. Lehmanns, Munich, Urban & Schwarzenberg, Vienna, Berlin, and after 1904 also Springer, Berlin, developed independent medical publishing programs with journals and books (cf. Jäger, pp. 473–483 [40]). In addition to universal journals, specialized journals were founded to publish original works like case studies, reports on new treatment methods and later clinical studies. During this time, case statistics from individual hospitals were also popular, as they offered a good overview of the clinical implementation of new therapies and surgical methods.

The development and demarcation of medical disciplines at the end of the 19th century led to the establishment of outpatient specialty clinics and new hospital departments. Curriculums for specialty training in Germany were just beginning to emerge. “Die dynamische Entwicklung unseres Fachgebietes in mehr als 100 Jahren spiegelt die stetige Suche nach dessen Stellenwert im Zusammenspiel mit den benachbarten Fachgebieten wider” (The dynamic development of our subject area in more than 100 years reflects the constant search for its place and significance, especially in respect to its neighbouring fields) [92]. Numerous new journals founded during this time therefore had the addition “… und ihre Grenzgebiete” (… and their associated fields). Because of the increasing number of journals in the same field, it was no longer possible for the practicing physician to keep up-to-date with all the new developments in his field. An abstract journal, often referred to as a “Zentralblatt”, was often created to meet this need. At the beginning of the 20th century, Springer Verlag in particular established a whole system of medical report organs which exclusively published medical reports, conference publications and reviews (cf. Sarkowski, p. 180 [73]). The *Internationale Zentralblatt für Laryngologie, Rhinologie und verwandte Wissenschaften* was established by Verlag Hirschwald, Berlin, as early as 1884. This was later continued as the *Zentralblatt Hals-Nasen-Ohrenheilkunde, plastische Chirurgie an Kopf und Hals, Organ der Deutschen Gesellschaft für Hals-Nasen-Ohrenheilkunde, Kopf- und Halschirurgie* as a conference publication organ and was discontinued in 1996 by Springer Verlag.

The classic universal journal is the *Deutsche Medizinische Wochenschrift* (DMW), which is still published today. It was founded by Paul Börner (1829–1885) in the Georg Reimer publishing house in 1875, and continued from 1887 onwards in Thieme Verlag. During this time, it was influenced by Rudolf Virchow who wrote on the topics of medical reform, hygiene and cellular pathology. It was also considered the house journal of Robert Koch (1843–1910) and Emil von Behring (1854–1917) because of their publications on the topics of cholera, diphtheria and tetanus [89]. Another universal journal that is still published is the *Archiv für pathologische Anatomie und Physiologie und klinische Medizin (Virchows Archiv)*, founded in 1847 by Rudolf Virchow and Benno Reinhardt (1819–1852). Usually, takeovers of publishers were based solely on economic motives of the publishers. In the case of Virchow’s archive, however, it was taken over by Springer Verlag in 1920 at the urging of the editors. Until then the journal had experienced declining subscriptions and been looked after by a “kleinlichen Prokuristen” (petty authorized signatory) of Georg Reimer Verlag (later Walter de Gruyther Verlag) in Berlin (cf. Sarkowski, p. 252 [73]). Overall, the first half of the 20th century was characterized by numerous takeovers of publishing houses, mergers of journals and a consolidation of the medical science journal market.

A special development took place around the turn of the last century with the spread and use of the German language as a scientific language. In the beginning, German was still widely used in academia as a lingua franca for nonnative speakers of German. German was often used as the language of international conventions and served as the translation language for international units. Scientists from Scandinavia or the Netherlands, for example, naturally published in German and in Eastern Europe, or even in Russia, German-language scientific journals appeared. At that time, German was an international scientific language on par with English. It was not until the aftermath of the two world wars that German lost its prominence as an international scientific language. Due to the economic ruin of the German-speaking countries and the loss of large parts of the Habsburg countries in Eastern Europe, in which German was the official language, German was used less and less as a lingua franca. The policy of the expulsion and murder of Jewish scientists and intellectuals by the Nazi regime also led to a dramatic loss of scientific potential in Germany (cf. Ammon 2001 [2]).

## Journal chronicles and the role of excellent scientists and publishers in founding new journals

With the development of medicine as a natural science at the beginning of the 19th century, the need for scientific exchange increased and an increasingly economically oriented market for publications emerged. The number of doctors and medical students in the German Reich increased significantly during this time. This led, on the one hand, to numerous new journals being founded by outstanding scientists. On the other hand, publishers became increasingly interested in founding new journals with a wide variety of layouts, publication intervals, content, categories and target groups. Not all journals were able to hold their own in the market. Some were discontinued; some were taken over by other publishers or merged with other journals. However, individual journals and publishers still exist today and have achieved outstanding national and international importance.

A look at today’s German-language ENT journals published by German publishers shows that most of these journals go back to foundations in the second half of the 19th and the first half of the 20th century. The relationship between publishers, editors and scientific societies is subject to constant change up to this day. Because some journals function as publication organs of the scientific medical societies in Germany, Austria and Switzerland, the journal’s success was linked to the scientific importance of these societies. Some were discontinued, some merged and some continued to be published for many decades. This can be observed today in the three-digit numbering of some journal volumes, like the current volume of the *European Archives of Oto-Rhino-Laryngology and Head & Neck* from 2020.The journal was founded in 1864 and is the world’s oldest journal devoted exclusively to an area of what would later become ear, nose and throat medicine, the *Archiv für Ohrenheilkunde*.

Title changes, discontinuations and mergers of the German-language scientific ENT journals make an historical overview somewhat confusing at first glance. In this section the attempt will be made to present the complex historical development of the German-speaking ENT journals as clearly as possible. Research on the development of journals was based primarily on library databases that were freely accessible on the web, such as WorldCat (www.worldcat.org), Hathitrust (www.hathitrust.org) or the ZDB journal database of the German National Library and the Berlin State Library (www.zdb-catalog.de). Every literature search is necessarily subjective and dependent on factors like the accessibility of literature, whether in traditional libraries or for free online, and the amount of time and effort expended. The present overview does not claim to provide the costly and extensive literature search necessary, for example, to create scientific guidelines. It is, rather, a cursory historical overview from a clinician’s point of view in 2020.

For a better overview, the journal titles in the tables are numbered in the order in which they are mentioned in the text, not chronologically. During the research, it turned out that the numbering of the volumes is helpful for orientation through mergers and acquisitions of journal titles, since it remains stable. The journal volumes are therefore shown in bold print. The aims and contents of journals can roughly be assessed in retrospect by looking at journal titles and title changes. Before ENT was fused to a speciality, early journals focused on individual organ systems such as the ear, the nose or the larynx. “Archives”, “Zentralblatt” or “Zeitschrift” in the title indicate a focus on original papers, reviews of papers, books or conferences. In contrast, the journal of the American Laryngological, Rhinological and Otological Society, Inc. with the title *The Laryngoscope* refers to only one organ area in the speciality. In addition, the importance of the mirror investigation is said to have served as the basis for the name of the journal founded in 1896: “So, the journal is dedicated to serve as an illuminating instrument in the continuing examination of the diseases we treat in our specialty” [6].

From the start, medical-scientific publishers have endeavoured to find suitable scientists and physicians as editors to shape the character and objectives of journals and recruit qualified authors. Publishers also tried to motivate famous scientists to participate in book projects and editorships in the hope that their reputation would reflect favourably on them and boost their economic success. While the publisher provides the entrepreneurial service and bears the economic risk, the editor is responsible for compiling contributions in encyclopedias, journals and serial publications [58]. The editors not only act as ambassadors for a journal, but are also responsible for the quality of the content and thus in turn contribute to its economic success. A literature search in publisher’s accessible sources reveals a large number of journal foundations, renaming and mergers [11, 32, 33, 45, 47, 73, 86, 89]. While foundations and mergers are usually for business reasons, renaming is often for content reasons. The emergence of ear, nose and throat medicine as a unified speciality from the end of the 19th to the beginning of the 20th century provides some examples. In 1915, for example, the *Archiv für Ohrenheilkunde*, predecessor of the current *European Archives of Oto-Rhino-Laryngology and Head & Neck*, was renamed *Archiv für Ohren‑, Nasen- und Kehlkopfheilkunde*, thus changing the title focus from one organ (the ear) to the whole field of ear, nose and throat. This coincided with the merger of ear clinics and clinics for laryngology and rhinology in German university hospitals. Furthermore, it became the publication organ for the German Society of Otolaryngologists, which was founded in 1921.

Editorships of journals change more frequently than the titles and changes of editor are often not shown in the library databases. In addition, naming the editor in the imprint is not mandatory even today (cf. Dernbach 2018 [13]). This makes it difficult to reconstruct editorships over time. In the journal database for Germany and Austria (ZDB), only the “issuing body”, i.e. the professional society is recorded. Biographical issues pertaining to journal editors, however, are of interest in the present work. Therefore it was necessary to inspect each print volume by hand and record the editor’s names. First names were often incomplete and had to be researched additionally along with important biographical information.

The term “editor” is not clearly defined either journalistically or legally. In today’s publishing industry, the editor is the link between the publisher and the editorial staff [13]. An editorial board consisting of distinguished personalities is often listed, but the creative tasks are carried out by only one or a few members of this group, e.g. the managing editor or editor in chief. In addition, some journals also have column or section editors and sometimes a scientific advisory board. In today’s publishing, editorial meetings define the technical and strategic direction of the journal in collaboration with the specialist scientific societies, and make personnel decisions regarding editorial board membership. The publisher also has a say, but generally sees itself as a service provider for the editor. There is now a trend towards a decline in print subscriptions, so that some English-language journals in particular are only published online. Some German-language medical science journals are not listed in the Medline database or in the Social Sciences Citation Index (SSCI). In these databases, which are dominated by US institutions and companies, hardly any non-English-language publications are listed, so that a bias in research in certain areas can be assumed.

Another problem is the so-called journal crisis that began in the 1990s. Consolidation processes in the scientific publishing market led to significant price increases for journals, creating considerable strains for academic libraries. The fact that science publishing houses generate large profits from publicly funded research has been criticized. This was followed by a boycott of some of the major science publishers by a number of library associations. As a result, the “Open Access” movement emerged, which aims to guarantee free access to scientific literature including primary and metadata on the Internet. The establishment of Open Access journals has been promoted in various European countries. In Germany for example, the “German Medical Science” portal was created. Various business models have been established in the meantime. Sometimes the author contributes to the publication costs by paying a publication fee. In institutional membership models, research institutions pay an annual fee to publish their research results in an open access journal. Open Access publications are increasingly being read, but have not yet been able to displace traditional journals in the impact-factor-generating databases. The establishment of economically focused “predator journals” with questionable business practices, aggressive advertising and inadequate peer review processes has also become problematic (cf. [103, 104]).

The “DEAL” project of the Alliance of German Science Organizations is taking an innovative approach. On the one hand, it facilitates access to electronic journals nationwide through new contract models with major science publishing houses such as Springer Nature, and on the other hand, it enables the authors of the participating scientific institutions to use the journals of the participating publishing houses to publish “Open Access”. For Springer Nature, this applies to the ENT journals *HNO *and* European Archives of Oto-Rhino-Laryngology and Head & Neck* [57].

Journal foundations, especially if they are still published today, are highlighted in chronological order below. Founding editors and publishers will be mentioned in their historical, academic and personal context. In the tabular compilation of the journals, all editors are initially listed, but in later cases, only the editors in chief are mentioned because of their significant historical influence on the design and direction of the respective journals. The historical sequence of the journals is shown in the tables. If the title of the journal changed or if the publisher changed due to a merger or sale, the continuation of the journal is usually visible in the numbering of the volumes. In some cases, however, the volume numbers were not continued. References to the previous journals were then found in editorials. From a current perspective in 2021 it is perhaps easiest to trace the history of journal foundations by looking at journals that are currently still in circulation. Therefore, the current publication organs of the German-speaking scientific societies will be presented first.

### Title history *European Archives of Oto-Rhino-Laryngology and Head & Neck*

#### *Official journal of the European Federation of Oto-Rhino-Laryngological Societies (EUFOS); official journal of the European Laryngological Society,* Berlin; Heidelberg: Springer [23]

On the occasion of the 150th anniversary of the founding of the *Archiv für Ohrenheilkunde* (*AfO*), the Leopoldina National Academy of Sciences in Halle/Saale held an international conference from May 7–10 in 2014. The *AfO* is the world’s oldest journal devoted exclusively to an area of what would later be ear, nose and throat medicine and still exists today as the *European Archives of Oto-Rhino-Laryngology and Head & Neck* [3, 65].

As shown in Table 1 (Journal Nos. 1, 7, 11 and 12), the European Archives go back to three journals founded in the 19th century: *Archiv für Ohrheilkunde* (1864), *Archiv für Augen- und Ohrenheilkunde* (1869) and *Archiv für Laryngologie und Rhinologie *(1893). The latter two were merged in 1922 to form the *Zeitschrift für Hals‑, Nasen- und Ohrenheilkunde* (1922). The *European Archives of Oto-Rhino-Laryngology and Head & Neck* is one of the oldest ENT journals in the world. In 1864, the journal was founded as *Archiv für Ohrenheilkunde* (*AfO*) in Würzburg in the publishing house of Stahel’s book and art dealers. By following the numbering of the volumes, the history of the journal can be traced, including changes in publishing houses and assumption of publishing houses with their journals and book titles. After the title was moved to the Leipzig publishing house F. C. W. Vogel in 1873, the name was changed to *Archiv für Ohren‑, Nasen- und Kehlkopfheilkunde *in 1915, and in 1922 the Vogel Verlag was taken over by Springer Verlag. After various changes to the title of the journal, it is still published today as volume 277 by Springer Nature as the *European Archives of Oto-Rhino-Laryngology and Head & Neck*.

**Table 1:** Title history and editors of *European Archives of Oto-Rhino-Laryngology and Head & Neck* [23]

1	***Archiv für Ohrenheilkunde***
	Leipzig: Vogel, Würzburg: Stahel [1864–1870] **1**. 1864 – **6**. 1873; N. F. (not followed) 1[= **7**].1873 – 4 = **10**. 1875/76; **11**. 1876 – **97**. 1914/15, continued as: Archiv für Ohren‑, Nasen- und Kehlkopfheilkunde
	Editors (1864–1915): Adam Politzer, Vienna, Hermann Schwartze, Halle, Anton von Tröltsch, Würzburg, Friedrich Kretschmann, Magdeburg, Paul Manasse, Strasbourg
2	***Archiv für Ohren‑, Nasen- und Kehlkopfheilkunde: Organ der*** ***Deutschen Gesellschaft der Hals‑, Nasen‑, Ohrenärzte***
	Berlin; Heidelberg; Göttingen: Springer **98**. 1915/16 – **154**. 1944/45,3/4; **155** = 52.1947/49; **156**. 1949/50 – **185**. 1965, continued as: Archiv für klinische und experimentelle Ohren‑, Nasen- und Kehlkopfheilkunde
	Editors (1915–1945): Alfred Denker, Halle/Saale, Otto Voss, Frankfurt/M, Karl Wittmaack, Jena/Hamburg, Oskar Wagener, Göttingen, Johannes Zange, Jena, Alfred Güttich, Cologne, Karl Beck, Heidelberg, Otto Mayer, Vienna, Adolf Greifenstein, Königsberg, Siegfried Unterberger, Vienna, Kaarlo Y. A. Meurman, Helsinki
	Editors (1947–1965): Hermann Frenzel, Göttingen, Carl von Eicken, Berlin, Wilhelm Lange, Leipzig, Alfred Seiffert, Heidelberg, Otto Steurer, Hamburg, Johannes Zange, Jena, Gustav Hofer, Graz, Erhard Lüscher, Basel, Adolf Miehlke, Göttingen, Emil Schlander, Vienna, Kaarlo Y. A. Meurman, Helsinki, Siegfried Unterberger, Klagenfurt, Max Schwarz, Tübingen, Fritz Zöllner, Freiburg/Br.
3	***Archiv für klinische und experimentelle Ohren‑, Nasen- und Kehlkopfheilkunde***
	Berlin: Springer **186**. 1966 – **205**. 1973, continued as: Archives of Oto-Rhino-Laryngology
	Editors (1966–1973): Hermann Frenzel, Göttingen, Karl-Heinz Vosteen, Frankfurt/M, Fritz Zöllner, Freiburg/Br., Chlodwig Beck, Freiburg/Br., Heinrich Spoendlin, Zurich/Innsbruck, Konrad Fleischer, Hamburg/Giessen, Walter Messerklinger, Graz, Carl Rudolf Pfaltz, Basel, Max Schwarz, Tübingen, Franz Altmann, New York, Hans-Joachim Denecke, Heidelberg, Hans Engström, Uppsala/Gotheburg, Paul Henry Holinger, Chicago, Adolf Miehlke, Göttingen, Masanori Morimoto, Kyoto, Jürgen Tonndorf, New York, Horst L. Wullstein, Würzburg, Walter Schätzle, Göttingen, Harold F. Schuknecht, Boston/MA, Walter Kley, Mainz
4	***Archives of Oto-Rhino-Laryngology: Organ of the Deutsche Gesellschaft für Hals-Nasen-Ohren-Heilkunde, Kopf- u. Hals-Chirurgie*** ***=*** ***Archiv für Ohren‑, Nasen- und Kehlkopfheilkunde***
	Berlin; Heidelberg [et al.]: Springer **206**. 1974 – **246**. 1989, continued as: European Achives of Oto-Rhino-Laryngology
	Editors (1974–1989): Chlodwig Beck, Freiburg/Br., Heinrich Spoendlin, Zurich/Innsbruck, Fritz Zöllner, Freiburg/Br., John M. Frederickson, Toronto, Uwe Ganzer, Mannheim/Düsseldorf, Alan David Kornblut, Washington D.C.
5	***European Archives of Oto-rhino-laryngology: official journal of the European Federation of Oto-Rhino-Laryngological Societies (EUFOS)***
	Berlin; Heidelberg [et al.]: Springer **247**. 1990 – **261**. 2004,1
	Editors (1990–2004): Chlodwig Beck, Freiburg/Br., Uwe Ganzer, Mannheim/Düsseldorf, Alan David Kornblut, Washington D.C., Heinrich Spoendlin, Zurich/Innsbruck, Gordon B. Snow, Amsterdam, Jan Olofsson, Bergen
6	***European Archives of Oto-Rhino-Laryngology and Head & Neck: official journal of the European Federation of Oto-Rhino-Laryngological Societies (EUFOS)***
	Berlin; Heidelberg [et al.]: Springer **261**. 2004,2 –
	Editors (2004–): Uwe Ganzer, Düsseldorf, Gordon B. Snow, Amsterdam, Jochen Werner, Marburg, Jan Olofsson, Bergen, Oliver Sterkers, Paris, Manuel Bernal-Sprekelsen, Barcelona, Roland Laszig, Freiburg/Br., Marc Remacle, Luxembourg
**Other previous titles**	
7	***Archiv für Augen- und Ohrenheilkunde***
	Wiesbaden: Bergmann; Karlsruhe: Müller **1**. 1869/70 – **7**. 1878/79, continued as: Zeitschrift für Ohrenheilkunde
	Editors (1869–1879): Hermann Knapp, New York, Samuel Moos, Heidelberg, Ludwig Mauthner, Innsbruck/Vienna
8	***Zeitschrift für Ohrenheilkunde***
	Wiesbaden: Bergmann **8**. 1879 – **34**. 1899, continued as: Zeitschrift für Ohrenheilkunde mit besonderer Berücksichtigung der Rhinologie und der übrigen Grenzgebiete
	Editors (1879–1899): Hermann Knapp, New York, Samuel Moos, Heidelberg, Otto Körner, Rostock, Arthur Hartmann, Berlin, Urban Pritchard, London
9	***Zeitschrift für Ohrenheilkunde mit besonderer Berücksichtigung der Rhinologie und der übrigen Grenzgebiete***
	Wiesbaden: Bergmann **35**. 1899 – **54**. 1907, continued as: Zeitschrift für Ohrenheilkunde und für die Krankheiten der Luftwege
	Editors (1899–1907): Hermann Knapp, New York, Otto Körner, Rostock, Arthur Hartmann, Berlin, Urban Pritchard, London
10	***Zeitschrift für Ohrenheilkunde und für die Krankheiten der Luftwege***
	Wiesbaden: Bergmann **55**. 1908 – **82**. 1922, continued as: Zeitschrift für Hals‑, Nasen- und Ohrenheilkunde (12)
	Editors (1908–1922): Hermann Knapp, New York, Otto Körner, Rostock, Arthur Hartmann, Berlin, Urban Pritchard, London, Gustav Killian, Freiburg/Br., Carl von Eicken, Berlin, Friedrich Siebenmann, Basel, Alfred Denker, Halle/Saale.
11	***Archiv für Laryngologie und Rhinologie***
	Berlin: Hirschwald **1**. 1893/94 – **34**. 1921, continued as: Zeitschrift für Hals‑, Nasen- und Ohrenheilkunde (12)
	Editors (1893–1921): Bernhard Fränkel, Berlin, Georg Finder, Berlin, Ottokar Chiari, Vienna, Paul Gerber, Königsberg, Otto Kahler, Freiburg/Br., Gustav Killian, Berlin, Hans Neumayer, München, Otto Seifert, Würzburg, Gustav Spiess, Frankfurt/M., Markus Hajek, Vienna
12	***Zeitschrift für Hals‑, Nasen- und Ohrenheilkunde***/Gesellschaft Deutscher Hals‑, Nasen- und Ohrenärzte
	Berlin: Springer **1**. 1922 – **51**. 1944, absorbed in: Archiv für Ohren‑, Nasen- und Kehlkopfheilkunde (2)
	Editors (1922–1944): Otto Körner, Rostock, Carl von Eicken, Berlin, Georg Finder, Berlin, Karl Wittmaack, Jena, Wilhelm Lange, Leipzig, Julius Hegener, Hamburg, Ernst Oppikofer, Basel, Reinhard Perwitzschky, Breslau, Ery Lüscher, Basel, Bernhard Langenbeck, Bonn

**Fig. 1:**
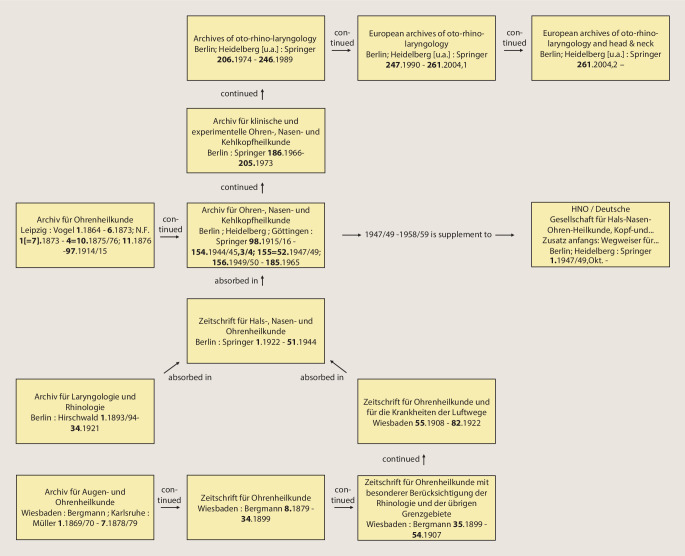
History of title changes and mergers of the *European Archives of Oto-Rhino-Laryngology and Head & Neck*

**Fig. 2:**
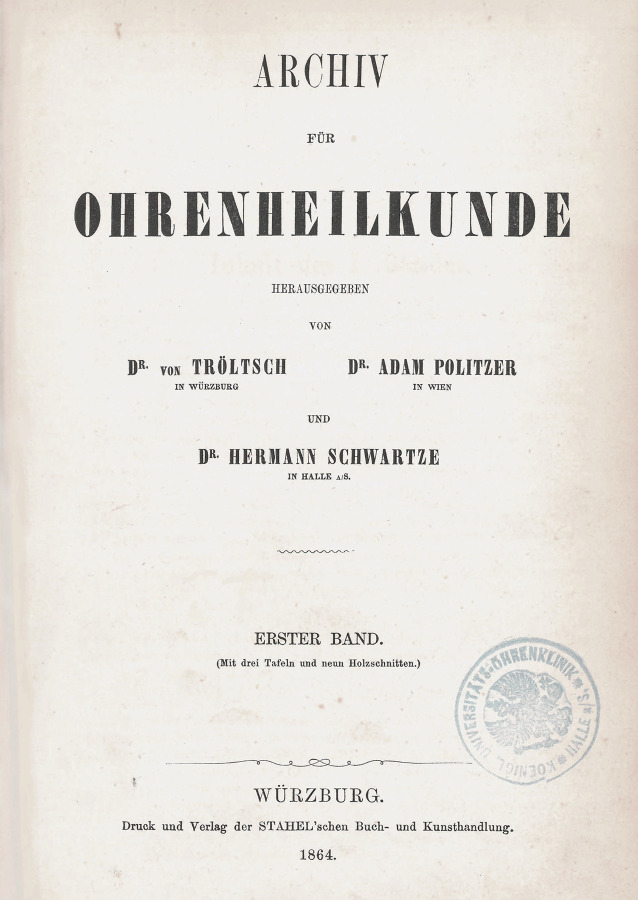
Title page of the first volume of the *Archiv für Ohrenheilkunde* from 1864

**Fig. 3:**
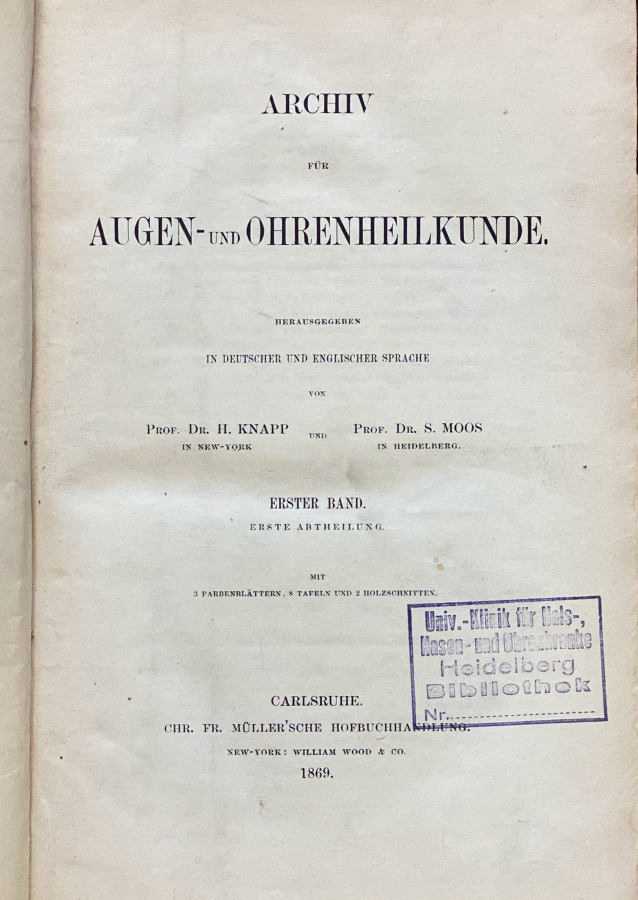
Title page of the first volume of the *Archiv für Augen- und Ohrenheilkunde* from 1869

**Fig. 4:**
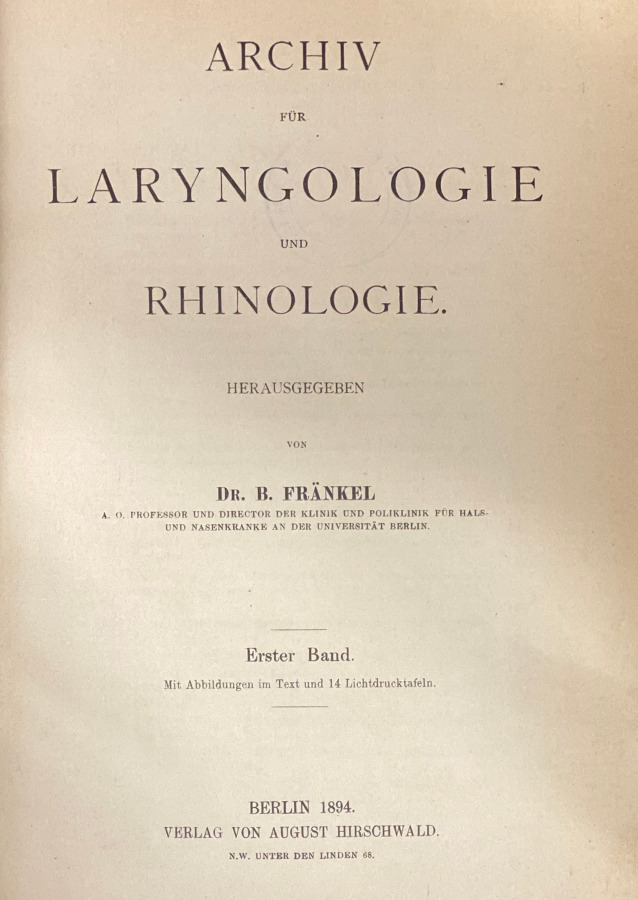
Title page of the first volume of the *Archiv für Laryngologie und Rhinologie* from 1894

**Fig. 5:**
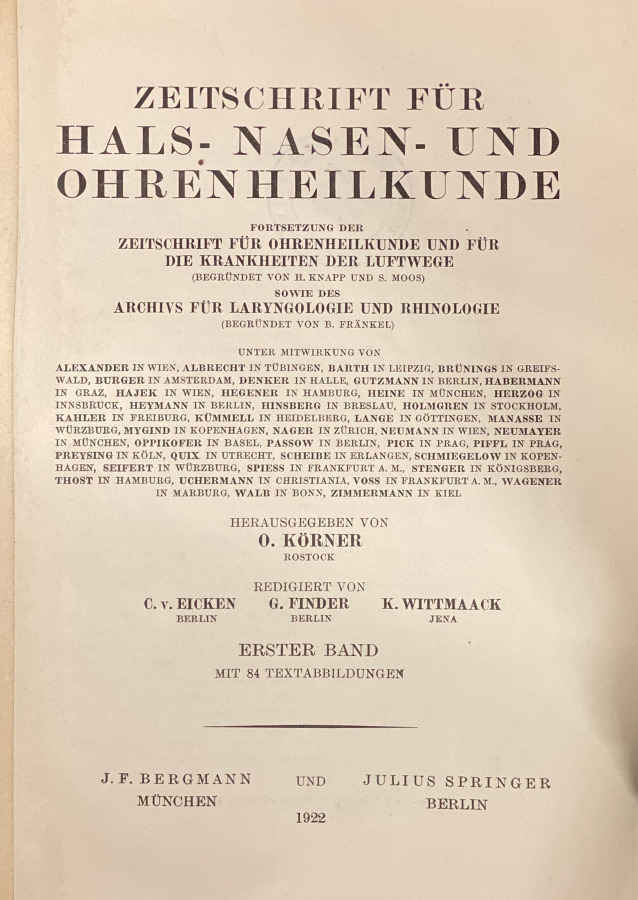
Title page of the first volume of the *Zeitschrift für Hals- Nasen- und Ohrenheilkunde* from 1922

Over the years, further journals were absorbed by the *AfO*, mainly through the purchase of the publishing houses Bergmann, Wiesbaden and Hirschwald, Berlin by Springer Verlag. This can also be easily seen in Table 1 based on the volume count: The *Archiv für Augen- und Ohrenheilkunde *(Bergmann) founded in 1869 went through various title changes and splits, and was finally sold to the Springer Verlag in 1922. Likewise, the *Archiv für Laryngologie und Rhinologie*, first published by Hirschwald in Berlin in 1893, came to Springer through a publisher sale in 1922. Both titles were merged to form the *Zeitschrift für Hals‑, Nasen- und Ohrenheilkunde *(*ZfHNO*). At the end of the Second World War, the *ZfHNO* was included in the *Archiv für Ohren‑, Nasen- und Kehlkopfheilkunde*, the successor of which is today’s *European Archives*. Figure 1 shows the course of title changes and mergers in a flow chart. The respective title pages of the newly founded predecessor journals of the *AfO* are shown in Figs. 2, 3, 4 and 5.

Most of the publishing houses known today emerged from bookshops with printing capacities in the 19th century. This is the case for the Stahel’sche Buch- und Kunsthandlung in Würzburg, which existed since 1753. For Anton von Tröltsch, a practicing ophthalmologist in Würzberg, who had recently completed his habilitation in the field of otology in 1861 [81] and the two other founding editors, Adam Politzer and Herrmann Schwartze, it was natural to found the *Archiv für Ohrenheilkunde* in collaboration with a local “publisher”. The establishment of the journal and the structure of its contents have already been reported elsewhere [62, 63]. The founding story is described in the same journal in 1890 by Schwartze in the obituary for Anton von Tröltsch and by Politzer on the 50th anniversary of the publication in 1914 as follows: “Das Interesse, welches durch die ersten Publikationen v. Tröltsch’s … für das bisher allgemein für unfruchtbar gehaltene Studium der Ohrenheilkunde erweckt war … führte zu der Idee der Begründung eines besonderen literarischen Organs, welches alles vereinigen sollte, was auf otologischem Gebiet gearbeitet wurde. Die erste Anregung ging (in einem Brief an v. Tröltsch vom 28. März 1863) von einem Augenarzte, Dr. Zander in Chemnitz aus …. Acht Monate später trat A. Politzer mit derselben Idee an v. Tröltsch heran” (The interest in the study of ear medicine, previously considered unprofitable, generated by Tröltsch’s first publications, … led to the idea of founding a special literary organ which would unite efforts in the otological field. The first suggestion came in a letter to von Tröltsch on March 28, 1863 from an ophthalmologist, Dr. Zander in Chemnitz …. Eight months later A. Politzer approached von Tröltsch with the same idea) [66, 81]. The first editors were Dr. Anton von Tröltsch from Würzburg, Dr. Adam Politzer from Vienna and Dr. Hermann Schwartze from Halle (Saale). Initially von Tröltsch had concerns about the viability of the journal. He feared insufficient staffing and a lack of customers. The journal foundation was only attempted after Hermann Schwartze committed to the project. Its success was later secured by the switch to the publishing house F. C. W. Vogel in Leipzig in 1873. When the journal was founded in 1864, all three editors were young men (von Tröltsch 35, Politzer 29 and Schwartze 27) with a great interest in ear medicine and were at the beginning of their medical and scientific careers. In the obituary for Anton von Tröltsch in 1890, Hermann Schwartze describes the difficult initial phase of the archive: “Die ersten Bände desselben folgten sich unregelmäßig und schleppend, und erst mit dem Übergang des Archivs in den Verlag von F. C. W. Vogel in Leipzig kam dasselbe in ein glattes Fahrwasser, als durch wachsende Zahl der Mitarbeiter die Beiträge reichlicher zuströmten und durch geregelten Geschäftsbetrieb des neuen Verlegers das schnellere Erscheinen der Arbeiten und die weitere Verbreitung des Archivs gesichert war” (The first volumes of the archive followed irregularly and slowly, and only with the transfer of the archive to the F. C. W. Vogel publishing house in Leipzig did it find its way into smooth waters. The growing number of employees made contributions more abundant and the regular business operations of the new publisher ensured the faster publication of the works and the wider distribution of the archive) (cf. Schwartze 1890, Mudry 2015 [62, 81]). In retrospect, it seems probable that the founders of the journal hoped for a wider distribution of the *AfO* in a large publishing company. The grandson of the publisher’s founder, Carl Victor Lampe-Vischer, had taken over the F. C. W. Vogel Verlag in 1862 and redirected the publishing program towards medicine (cf. Sarkowski, p. 312 [73]). Leipzig was Germany’s centre for books and publishing until World War II. The *AfO* was transferred in 1930, when the publisher F. C. W Vogel was sold to Springer. The journal volumes continue to follow the numbering as the English language *European Archives of Oto-Rhino-Laryngology and Head & Neck* under the editorship of Manuel Bernal-Sprekelsen, Roland Laszig and Marc Remacle.

In their 1869 editorial for the first issue of their newly founded journal *Archiv für Augen- und Ohrheilkunde*, Hermann Knapp (1832–1911), New York and Salomon Moos (1831–1895), Heidelberg, refer to the ground-breaking progress that came with the invention of the ophthalmoscope for the examination of the sensory organs, the eye and the ear. “Die nahe Verwandtschaft der Augen- und Ohrenheilkunde … fordert dazu auf, beide vereinigt zu betreiben” (the close relationship of ophthalmology and ear medicine … calls for both to be practiced together) [51]. Knapp, who completed his habilitation (postdoctoral lecturing qualification in Germany) under Helmholtz in Heidelberg in 1859, founded an eye clinic for inpatients and outpatients there in 1862. Because of the delay in the planned construction of his own university eye clinic in Heidelberg, Knapp emigrated to New York in 1868. In keeping with the American trend at the time, Knapp included the treatment of ear diseases when he founded his “New York Ophthalmic and Aural Institute” [54]. Together with Salomon Moos, associate professor for otology in Heidelberg since 1866, Knapp founded the journal *Archiv für Augen- und Ohrenheilkunde/Archives of Ophthalmology and Otology *in 1869, which was published in New York with William Wood in English and in Karlsruhe in German with Müller’sche Hofbuchhandlung. This connection between American and German medicine was not only due to the personal relationship between Moos and Knapp from their time together in Heidelberg, but was also an indication for the high regard for German speciality disciplines in the USA at that time because of their solid scientific foundations (cf. Koelbing 1979 [54]). The archive was continued in 1879 as the 8th volume of *Zeitschrift für Ohrenheilkunde* and *Archiv für Augenheilkunde* by Bergmann publishing.

Bernhard Fränkel (1836–1911) founded the *Archiv für Laryngologie und Rhinologie* in the Berlin publishing house August Hirschwald in 1893. The laryngologist Fränkel was a student of Rudolf Virchow. He completed his habilitation in 1887 and was appointed head of an independent institute for laryngology and rhinology at the Berlin Charité in 1887. Six years later he was appointed director of the newly established clinic for throat and nose patients, which he headed until shortly before his death in 1911. Subsequently, Georg Finder (1867–1931), senior physician at the Charité under Fränkel and his successor Gustav Killian, was editor of the journal until it was taken over by Springer Verlag in 1921. In addition to Finder, the laryngologists Ottokar Chiari (1853–1918), Vienna, Paul Gerber (1863–1919), Königsberg, Gustav Killian (1869–1921), Berlin, Markus Hajek (1861–1941), Vienna, Otto Kahler (1878–1946), Freiburg/Br., and Hans Neumayer (1865–1938), Munich, worked as co-editors.

The founding of new medical journals went hand in hand with the establishment of speciality medical societies. Between 1850 and 1914 alone, 65 medical and scientific societies were founded [68]. In 1892 the German Otological Society was founded, in 1894 the Association of South German Laryngologists and in 1905 the German Laryngological Society. It was therefore natural for corresponding journals to be founded at the same time. The takeover of Hirschwald Verlag by Springer in 1922 and the associated relocation of journals were mainly for family or business reasons. August Hirschwald opened a bookstore in Berlin in 1816 and started publishing in 1826. The publishing house August Hirschwald was very successful in the field of medicine early on, but at the turn of the century it faced very strong competition from the publishers F. C. W. Vogel, J. F. Bergmann, G. Fischer, S. Karger, G. Thieme and Urban & Schwarzenberg. After the First World War, Hirschwald publishing house was taken over by Springer [73]. Starting in 1922, the *Archiv für Laryngologie und Rhinologie* was continued as the *Zeitschrift für Hals‑, Nasen- und Ohrenheilkunde*, but with a new volume count.

### Title history *HNO*

#### *Deutsche Gesellschaft für Hals-Nasen-Ohren-Heilkunde, Kopf- und Hals-Chirurgie, Deutsche Akademie für Hals-Nasen-Ohren-Heilkunde, Kopf- und Hals-Chirurgie*, Heidelberg: Springer Verlag [25]

**Fig. 6:**
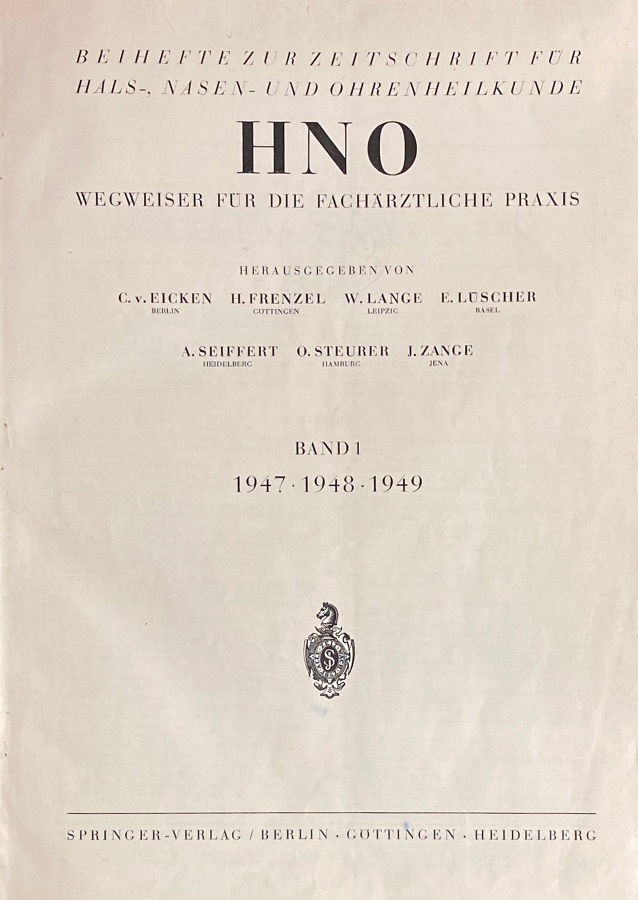
Title page of the first volume of the *HNO* from 1947/1948/1949

The title history of *HNO* is quite straightforward. It was founded by Springer Verlag after World War II, with a focus on practical topics. The journal *HNO/Organ der Deutschen Gesellschaft für Hals- Nasen- Ohrenheilkunde*, *Kopf- und Hals-Chirurgie* was founded by Springer Verlag in 1947 as a supplement to the *Zeitschrift für Hals-Nasen- und Ohrenheilkunde*. Although this journal had already been included in the *Archiv für Ohren‑, Nasen- und Kehlkopfheilkunde*, this addition can still be found on the cover. This is most likely due to the recommencement of the publishing activities after the World War II with initial licensing difficulties in the western occupation zones. In a letter from 1947, Ferdinand Springer complains about the denial of a license for the most important and best-known journals of his publishing house, including the *Archiv für Ohren‑, Nasen- und Kehlkopfheilkunde (vereinigt mit Zeitschrift für Hals-Nasen-Ohrenheilkunde) *by American authorities (cf. Götze, 1994, p. 4 [33]). In 1958 the addition to the name was dropped and the title *HNO* has since appeared as the publication organ of the German Society for Otorhinolaryngology, Head and Neck Surgery and later also the German Academy for Otorhinolaryngology, Head and Neck Surgery under the same name. In the first few years, the *HNO* also had the additional title “Wegweiser für die fachärztliche Praxis” (Guide for specialist medical practice), which suggests the more clinical–practical objectives of the publisher and the first editor Hermann Frenzel (1895–1967). Frenzel, who completed his habilitation in ear, nose and throat medicine under Wilhelm Brünings in Greifswald in 1925, was appointed to Göttingen as a full public professor in 1942. In his obituary for Hermann Frenzel in 1968, Minnigerode describes the relationship between the *HNO* and the *Archiv* in the post-war period as follows: “Nach dem Rücktritt Seiferts, mit dem er sich gemeinsam um das Wiedererscheinen und die Neuorganisation unserer Fachzeitschrift durch Teilung in das der klinischen und experimentellen Forschung dienende Archiv und den auf die Fachpraxis ausgerichteten HNO-Wegweiser bemüht hatte, übernahm Hermann Frenzel 1955 die Schriftleitung des *Archivs für Hals-Nasen-Ohrenheilkunde*, dessen geschäftsführender Herausgeber er bis zu seinem Tode war” (After Seifert’s resignation, with whom he worked together to relaunch and reorganize our journal by dividing it into the *Archiv*, which served clinical and experimental research, and the ENT guide, which was aimed at specialist practice, Hermann Frenzel took over the editorship of the *Archiv* for ear, nose and throat medicine in 1955, and was its managing editor until his death) [61]. From the start, *HNO* was the publication organ of the German Society for Otorhinolaryngology, Head and Neck Surgery, the Swiss Society for Oto-Rhino-Laryngology, Neck and Facial Surgery (from 1977), and several regional ENT societies, including, interestingly, the East German medical societies for ENT medicine in Halle, Jena, Leipzig, Rostock and Greifswald until 1975. In 2008 *HNO* also became the new publication organ of the Austrian ENT Society. The common scientific and professional interests of the professional societies were influential in the development of ENT as a discipline in Europe [10]. Since 2016 *HNO* has been published not only in German, but also in English in a supplement volume that appears online twice a year. An exclusively English-language publication is also possible in exceptional cases. Table 2 lists the editors of *HNO* since 1947. Figure 6 shows the title page of the first volume of *HNO* from 1947/1948/1949.

**Table 2:** Title history and editors of *HNO* [25]

***HNO/Deutsche Gesellschaft für Hals-Nasen-Ohren-Heilkunde, Kopf- und Hals-Chirurgie, Deutsche Akademie für Hals-Nasen-Ohren-Heilkunde, Kopf- und Hals-Chirurgie***
Heidelberg: Springer Verlag GmbH, Springer Medizin; Heidelberg: Springer-Medizin-Verl. **1. **1947/49, Oct. –
**1. **1947/49 –** 7. **1958/59 supplement to: Archiv für Ohren‑, Nasen- und Kehlkopfheilkunde (2–Tab. 1)
Editors (1947–): Carl von Eicken, Berlin, Hermann Frenzel, Göttingen, Wilhelm Lange, Leipzig, Erhard Lüscher, Basel, Alfred Seiffert, Heidelberg, Otto Steurer, Hamburg, Johannes Zange, Jena (founding editor), Kaarlo Y. A. Meurman, Helsinki, Max Schwarz, Tübingen, Siegfried Unterberger, Klagenfurt, Julius Berendes, Marburg, Rudolf Link, Hamburg, Adolf Miehlke, Göttingen, Rudolf Pfaltz, Basel, Franz Escher, Bern, Ernst Lehnhardt, Hannover, Hans-Peter Zenner, Tübingen, Peter-Karl Plinkert, Heidelberg, Stefan Plontke, Halle/Saale

### Title history *Laryngo-Rhino-Otologie: LRO*

#### *Organ der Deutschen Gesellschaft für Hals-Nasen-Ohren-Heilkunde, Kopf- und Hals-Chirurgie; Organ der Deutschen Akademie für Hals-Nasen-Ohren-Heilkunde, Kopf- und Hals-Chirurgie; Organ der Österreichischen Gesellschaft für Hals-Nasen-Ohren-Heilkunde, Kopf- und Hals-Chirurgie* Stuttgart; New York, NY: Thieme 68. 1989 – [18]

**Fig. 7:**
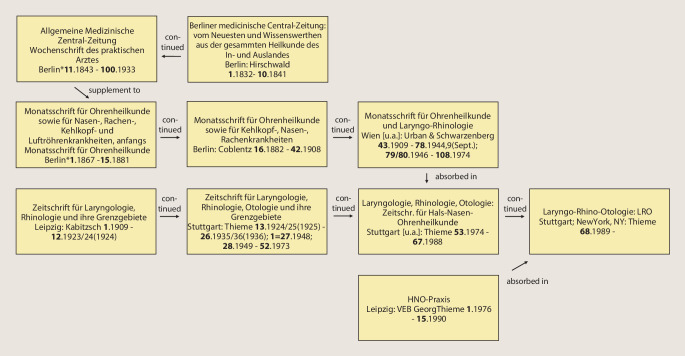
Course of the title changes and mergers of the *LRO (Asterisk *Expedition der *Allgemeinen Medizinischen Central-Zeitung*)

**Fig. 8:**
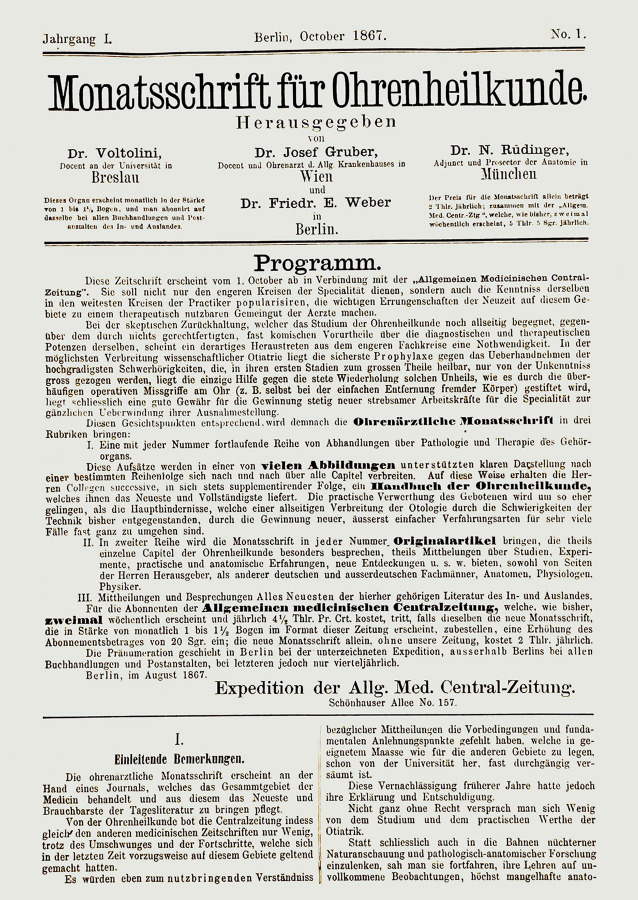
Title page of the first volume of *Monatsschrift für Ohrenheilkunde* from 1867

**Fig. 9:**
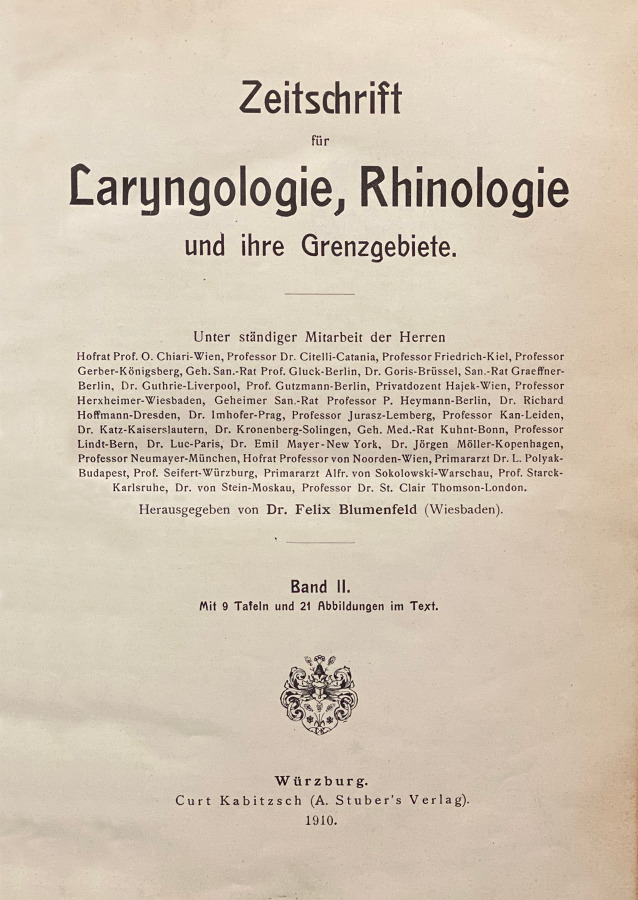
Title page of the first volume of *Zeitschrift für Laryngologie Rhinologie* *und ihre Grenzgebiete* from 1910

The journal *Laryngo-Rhino-Otologie: LRO*, published today by Thieme Verlag Stuttgart, New York, NY, reached volume number 99 in 2020. It goes back to two journals founded around 1900: the *Monatsschrift für Ohrenheilkunde* and the *Zeitschrift für Laryngologie, Rhinologie und ihre Grenzgebiete*. If one follows the volume numbers, the first volume was founded in 1909 in Leipzig by C. Kabitzsch as the *Zeitschrift für Laryngologie, Rhinologie und ihre Grenzgebiete*. This journal was issued as *Zeitschrift für Laryngologie, Rhinologie, Otologie und ihre Grenzgebiete: Organ d. Vereinigung Südwestdeutscher Hals‑, Nasen‑, Ohrenärzte *after World War II by Thieme in Stuttgart up to volume 52 in 1973. The second foundation dates back to 1867. The *Monatsschrift für Ohrenheilkunde sowie für Nasen‑, Rachen‑, Kehlkopf- und Luftröhrenkrankheiten*, initially *Monatsschrift für Ohrenheilkunde*, was published until 1881 as *Expedition der Allgemeinen Medizinischen Centralzeitung* in Berlin and from 1882 as *Monatsschrift für Ohrenheilkunde sowie für Kehlkopf‑, Nasen‑, Rachenkrankheiten: Organ d. Österreichischen Otologischen Gesellschaft u. d. Münchener Laryngo-Otologischen Gesellschaft*, continued by Oscar Coblentz Verlag, Berlin. From volume 43 in 1909, the publishing house Urban & Schwarzenberg in Vienna published it as *Monatsschrift für Ohrenheilkunde und Laryngo-Rhinologie: Organ der Österreichischen Oto-laryngologischen Gesellschaft und der Wiener Gesellschaft der Hals‑, Nasen‑, Ohren-Ärzte*. The latter journal retained its independence up to volume 108 and was incorporated into the journal *Laryngologie, Rhinologie, Otologie* by Thieme in Stuttgart in 1974. Figure 7 shows the course of title changes and mergers in a flow chart. The respective title pages of the newly founded predecessor journals of the *LRO* are shown in Figs. 8 and 9.

**Fig. 10:**
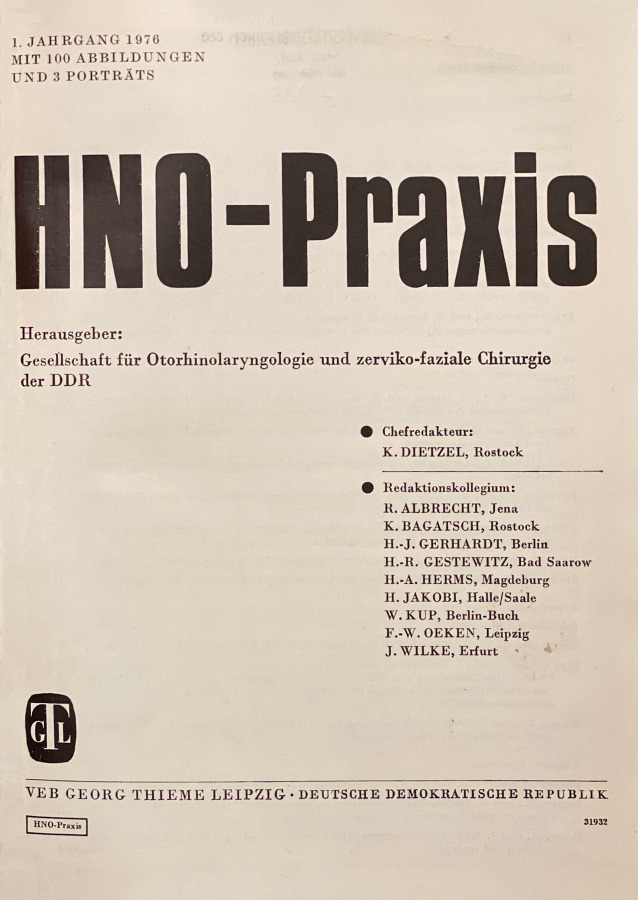
Title page of the first volume of the *HNO-Praxis* from 1976 (with kind permission from ©Georg Thieme Verlag KG, all rights reserved)

Initially, *Monatsschrift für Ohrenheilkunde* was founded as an “Expedition” (shipping issue) of the *Allgemeine Medizinische Central-Zeitung* in 1867 in Berlin. This universal journal was published from 1832 to 1933 by the Berlin publishing house Hirschwald as the *Berliner medicinische Central-Zeitung*, then by the founder of the journal, Johann Jakob Sachs (1803–1846), and later by the Berlin publishing house Oskar Coblentz [70]. At the time of the foundation of the monthly publication, the publishing house of the *Allgemeine Medizinische Central-Zeitung* belonged to the widow of the first editor, Fanny Sachs. The first editors of the monthly were Rudolf Voltolini (1819–1889), Breslau, Josef Gruber (1827–1900), Vienna, Nikolaus Rüdinger (1832–1896), Munich and Friedrich Eugen Weber, Berlin, from 1873 Weber-Liel (1832–1891). Weber had added his mother’s maiden name to his name for personal reasons. Rüdinger worked as an anatomist in Munich. The three otologists had completed their habilitation in otology around the time the journal was founded: Voltolini in 1862, Gruber in 1863 and Weber in 1872. In the editorial to the first volume for the new journal, the editors describe three goals. Founded as a supplement to a classic universal journal, the paper was intended to give practicing physicians an insight into the speciality of otology through illustrated articles. Furthermore, original articles were to underscore the scientific character of the journal and finally, reviews of the latest literature from home and abroad were to be provided (cf. Weber 1867 [101]). In the further historical development of scientific journals, these three functions have often been realized in separate journals, some focusing on clinical trials, continuous medical education and journal reviews (“Zentralblätter”). In the present, current journals such as *HNO* and *LRO* contain rubrics like “original scientific research”, “preparation for the specialist examination”, “CME” (continuing medical education) and “update”. The content of the journal, which had hitherto been predominantly otological, was expanded in 1876 and “Nasen‑, Rachen‑, Kehlkopf- und Luftröhrenkrankenheiten” (nose, pharynx, larynx and trachea diseases) was added to the title. This reflected increased scientific and clinical recognition of the close relationships between the organs. In France, this fusion took place a year earlier. In 1875 the *Annales des maladies de l’oreille et du larynx (otoscopie, laryngoscopie, rhinoscopie)* was founded, covering the whole spectrum of ENT.

The introduction of a general index in 1934 was a highlight in the history of the “Monatsschrift”. In today’s digital age, one can hardly imagine the effort necessary to create the index by hand. Since the journal was initially published by other publishers, the Urban & Schwarzenberg publishing house decided to record the central index from volume 43 of 1909 to volume 67 of 1933. The index contained not only author names but also subjects (cf. Urbantschitsch 1934 [96]). Other journals, such as *AfO*, also kept extensive general indexes during this period [37]. On the occasion of the 100th anniversary of the “Monatsschrift” in 1966, Fremel commented on the important function of the journal as a unifying factor in multi-ethnic Austria-Hungary. The “Monatsschrift” was no longer able to fulfill this specifically Austrian role after the German Reich co-ordination in 1938 (cf. Fremel 1966 [31]). After the founding of the Austrian Otolaryngeological Society in 1945/46 under its President E. Urbantschitsch, the journal appeared again in its old form (cf. [31]). The “Monatsschrift” was continued under the title *Monatsschrift für Ohrenheilkunde und Laryngo-Rhinologie* up to volume 108, 1974, at the publishing house Urban & Schwarzenberg and from 1974 onwards it appeared in the journal *Laryngologie, Rhinologie, Otologie* of the Stuttgart-based Thieme Verlag. However, the official addition “… vereinigt mit Monatsschrift für Ohrenheilkunde” (… united with the monthly journal for otology) only appears in the *LRO* only in 1976 from volume 55. There are no references to the reasons for the absorption of “Monatsschrift” by *LRO* in the editorials of either journal at the time, nor is it reported in the publisher’s anniversary publications. It can be assumed that the transition of the “Monatsschrift” from Verlag Urban & Schwarzenberg to Thieme Verlag was for economic reasons. From 1909 to 1974 a total of 17 publishers successively directed the journal (Table 3).

**Table 3:** Title history and editors of *Laryngo-Rhino-Otologie: LRO* [18]

1	***Monatsschrift für Ohrenheilkunde sowie für Nasen‑, Rachen‑, Kehlkopf- und Luftröhrenkrankheiten, initially Monatsschrift für Ohrenheilkunde***
	Berlin: Expedition der Allgemeinen Medizinischen Zentralzeitung **1**. 1867 – **15**. 1881
	Editors (1867–1881): Rudolf Voltolini, Breslau, Josef Gruber, Vienna, Nikolaus Rüdinger, München, Friedrich Eugen Weber, Berlin (founding editor), Leopold von Schrötter, Vienna, Max-Joseph Oertel, München
2	***Monatsschrift für Ohrenheilkunde sowie für Kehlkopf‑, Nasen‑, Rachenkrankheiten: Organ der Österreichischen Otologischen Gesellschaft u. d. Münchener Laryngo-Otologischen Gesellschaft***
	Berlin: Coblentz **16**. 1882 – **42**. 1908
	Editors (1882–1908): Rudolf Voltolini, Breslau, Josef Gruber, Vienna, Nikolaus Rüdinger, München, Friedrich Eugen Weber, Berlin, Leopold von Schrötter, Vienna, Max-Joseph Oertel, München, Michael Joseph Rossbach, Würzburg, Philip Schech, München, Emil Zuckerkandl, Vienna, Viktor Urbantschitsch, Vienna, Antoni Jurasz, Heidelberg
3	***Monatsschrift für Ohrenheilkunde und Laryngo-Rhinologie: Organ der Österreichischen Oto-laryngologischen Gesellschaft und der Wiener Gesellschaft der Hals‑, Nasen‑, Ohren-Ärzte***
	Vienna [u. a.]: Urban & Schwarzenberg **43**. 1909 – **78**.1944,9(Sept.); **79/80**. 1946 – **108**. 1974, absorbed in: Laryngologie, Rhinologie, Otologie
	Editors (1909–1944): Emil Zuckerkandl, Vienna, Viktor Urbantschitsch, Vienna, Antoni Jurasz, Lemberg, Ottokar Chiari, Vienna, Heinrich Neumann, Vienna, Markus Hajek, Vienna, Gustav Alexander, Vienna, Gustav Hofer, Graz, Wilfried Krainz, Innsbruck, Hermann Marschik, Vienna, Ernst Urbantschitsch, Vienna, Siegfried Unterberger, Vienna, Emil A. Wessely, Vienna
	Editors (1946–1974): Ernst Urbantschitsch, Vienna, Otto Novotny, Vienna, Friedrich Krejci, Vienna, Eduard H. Majer, Vienna, Kurt Burian, Vienna
4	***Zeitschrift für Laryngologie, Rhinologie und ihre Grenzgebiete***
	Leipzig: Kabitzsch **1**. 1909 – **12**. 1923/24(1924), continued as: Zeitschrift für Laryngologie, Rhinologie, Otologie und ihre Grenzgebiete
	Editors (1909–1924): Felix Blumenfeld, Wiesbaden
5	***Zeitschrift für Laryngologie, Rhinologie, Otologie und ihre Grenzgebiete: Organ d. Vereinigung Südwestdeutscher Hals‑, Nasen‑, Ohrenärzte***
	Stuttgart: Thieme **13. **1924/25(1925) – **26. **1935/36(1936); **1** **=** **27**. 1948; **28. **1949 – **52. **1973, continued as: Laryngologie, Rhinologie, Otologie
	Editors (1924–1936): Felix Blumenfeld, Wiesbaden, Alexander Herrmann, Erfurt
	Editors (1948–1973): Hans Leicher, Stuttgart/Mainz, Max Meyer, Würzburg, Richard Mittermaier, Marburg/Frankfurt/M, Gerhard Theissing, Ludwigshafen/Erlangen, Walter Becker, Bonn, Hans-Georg Boenninghaus, Heidelberg
6	***Laryngologie, Rhinologie, Otologie: Zeitschr. für Hals-Nasen-Ohrenheilkunde; Organ der Deutschen Gesellschaft für Hals-Nasen-Ohren-Heilkunde, Kopf- und Halschirurgie; Organ der Österreichischen Gesellschaft für Hals-Nasen-Ohren-Heilkunde, Kopf- und Halschirurgie***
	Stuttgart [u. a.]: Thieme **53**. 1974 – **67**. 1988, continued as: Laryngo-Rhino-Otologie
	Editors (1974–1988): Hans-Georg Boenninghaus, Heidelberg, Hans-Heinz Naumann, München, Kurt Burian, Vienna, Harald Feldmann, Münster
7	***Laryngo-Rhino-Otologie: LRO: Organ der Deutschen Gesellschaft für Hals-Nasen-Ohren-Heilkunde, Kopf- und Hals-Chirurgie; Organ der Deutschen Akademie für Hals-Nasen-Ohren-Heilkunde, Kopf- und Hals-Chirurgie; Organ der Österreichischen Gesellschaft für Hals-Nasen-Ohren-Heilkunde, Kopf- und Hals-Chirurgie***
	Stuttgart; New York, NY: Thieme **68**. 1989 –
	Editors (1989–): Harald Feldmann, Münster, Kurt Burian, Vienna, Ernst Kastenbauer, München, Heinz Stammberger, Graz, Heinrich Rudert, Kiel, Jan Helms, Würzburg, Gerhard Rettinger, Ulm, Orlando Guntinas-Lichius, Jena, Andreas Dietz, Leipzig, Stefan Dazert, Bochum, Gerd Rasp, Salzburg

**Table 4:** Title history and editors of *HNO-Praxis/Hrsg.: Gesellschaft für Oto-rhino-laryngologie und Zerviko-faziale Chirurgie der DDR* [20]

***HNO-Praxis/Hrsg.: Gesellschaft für Oto-rhino-laryngologie und Zerviko-faziale Chirurgie der DDR***
Leipzig: VEB Georg Thieme **1. **1976 – **15. **1990
Editors (1976–1990): Kurt Dietzel, Rostock, Hans-Jürgen Gerhard, Berlin, Lutz Kessler, Dresden

**Table 5:** Title history and editors of *ORL: journal for oto-rhino-laryngology *[19]

1	*Beiträge zur Anatomie, Physiologie, Pathologie und Therapie des Ohres, der Nase und des Halses*
	Berlin: Karger **1**. 1908 – **24**. 1926
	Editors (1908–1926): Adolf Passow, Berlin, Karl Ludolf Schaefer, Berlin
2	*Passow-Schäfer Beiträge zur Anatomie, Physiologie, Pathologie und Therapie des Ohres, der Nase und des Halses*
	Berlin: Karger **25**. 1927 – **31**. 1934/35[?]
	Editors (1927–1935): Alfred Güttich, Greifswald, Karl Ludolf Schaefer, Berlin, Oskar Wagener, Göttingen, Johannes Zange, Jena
3	*Practica oto-rhino-laryngologica: internat. Zeitschrift für Hals‑, Nasen‑, Ohrenheilkunde*
	Basel [et al.]: Karger **1**. 1938 – **33**. 1971,6
	Editors (1938–1971): Joseph Berberich, Frankfurt/M/New-York NY, Emil Schlittler, Basel, Georges Canuyt, Strasbourg, Georg Kelemen, Budapest, Robert Lund, Kopenhagen, Vincenco Tanturri, Mailand (founding editor), Luzius Rüedi, Zurich, Eelco Huizinga, Groningen, Carl Rudolf Pfaltz, Basel
4	*ORL: journal for oto-rhino-laryngology and its related specialities*
	Basel; Freiburg, Br.; Paris; London; New York; New Delhi; Bangkok; Singapore; Tokyo; Sydney: Karger **34**. 1972–
	Editors (1972–): Carl Rudolf Pfaltz, Basel, Wolfgang Arnold, Luzern/München, Bert W. O’Malley, Jr. Philadelphia, PA, Daqing Li, Philadelphia, PA

The *Zeitschrift für Laryngologie, Rhinologie und ihre Grenzgebiete* was founded in 1909 by Curt Kabitzsch publishing house in Würzburg. A look at the numbering of the volumes shows that it is continued to this day (2020) with volume 99 as *LRO* at Thieme Verlag, Stuttgart. The founding editor was Felix Blumenfeld (1864–1947) from Wiesbaden. In the editorial to the first volume, the editor refers to the introduction of the laryngeal mirror by Türck and Czermak 50 years earlier. The importance of this innovation was highlighted at the first international laryngologist congress in Vienna in 1908. The introduction of laryngoscopy into clinical practice is recognized as a great achievement and as the beginning of scientific laryngology (cf. Blumenfeld 1909 [9]). The new journal helped to further expand this field and meet the needs of the practical ear, nose and throat doctor. For decades, Blumenfeld ran a large specialist medical practice in the spa town of Wiesbaden, and was well-known far beyond the borders of his home region for his extensive journalistic activities. He was co-editor of several textbooks on surgery, pathology and tuberculosis. Until the National Socialists revoked his editorial status in 1934, he was the uninterrupted editor of the *Zeitschrift für Laryngologie, Rhinologie und ihre Grenzgebiete* (cf. editor and publisher 1948 [80]). The only change was the addition of the term “Otologie” (otology) to the title of the journal in 1925, which mirrored the unification of the specialist field by then. The considerable reputation of Blumenfeld and the journal is recognizable in the collaboration of numerous professional representatives from home and abroad mentioned on the respective covers. Although it was not possible to find clear sources of information on the withdrawal of Felix Blumenfeld’s editorship and comments on this, it can be assumed that the international reputation of German ENT and that of the publisher clearly suffered as a result. The Curt Kabitzsch Verlag, which was taken over by the Leipzig-based publishing house J.A. Barth in 1917, changed the name of the journal to *Folia oto-laryngologica* under the editorship of Alexander Herrmann, Erfurt (1900–1981). The last pre-war volume was published in 1936 as number 26 part 1. After the war *Zeitschrift für Laryngologie, Rhinologie Otologie und ihre Grenzgebiete* appeared in the Thieme publishing house as a new foundation, initially as volume 1. Later, this volume was given the number 27, following the numbering of the earlier journal *LRO* [98]. This can be explained by the licensing procedures of occupation authorities in 1948. The first editors after the war were Hans Leicher (1898–1989), Stuttgart, Max Meyer (1890–1954), Würzburg, Richard Mittermaier (1897–1983), Marburg, Gerhard Theissing (1903–1987), Ludwigshafen/Erlangen and Walter Becker (1920–1990), Bonn. From volume 53 on, published in 1971, the editors were listed on the cover in addition to editorial board and advisory board. Long-time editors were Hans-Georg Boenninghaus (1921–2005), Heidelberg, Hans-Heinz Naumann (1919–2001), Munich, Kurt Burian (1921–1996), Vienna, Harald Feldmann (1926–), Münster, Ernst Kastenbauer (1937–2004), Munich, Heinz Stammberger (1946–2018), Graz, Heinrich Rudert (1935–), Kiel, Jan Helms (1937–), Würzburg, Gerhard Rettinger (1947–), Ulm and Orlando Guntinas-Lichius (1967–), Jena. The current editor in chief of *LRO*, Andreas Dietz (1962–), Leipzig, is supported by 3 further editors (Stefan Dazert (1963–), Bochum, Orlando Guntinas-Lichius, Jena, Gerd Rasp (1960–), Salzburg), 11 section editors, 29 editors, 11 editores emeriti and 8 advisory board members.

### Title history *HNO-Praxis*

#### Gesellschaft für Oto-rhino-laryngologie und Zerviko-faziale Chirurgie der DDR Leipzig: VEB Georg Thieme [20]

The history of *HNO-Praxis* is quite straightforward (Table 4). Founded in 1976 at VEB Georg Thieme Leipzig publishing house, the title *Folia broncho-oesophagologica* was added in 1980 (Fig. 10). After the reunification of Germany in 1990, the journal was absorbed in *LRO*.

Because of the increasing number of ENT specialists in the German Democratic Republic (GDR) and the growing scientific potential in the university clinics, a scientific ENT journal was established on behalf of the General Secretariat of the Medical Scientific Societies at the Ministry of Health of the GDR and at the request of the Society for Otorhinolaryngology and Cervico-Facial surgery of the GDR in 1976. In 2000, Scholz described the special situation of medicine in the GDR at that time as follows: “Die Maßnahmen für eine Stärkung der DDR mit ständig steigender Abgrenzung gegenüber der Bundesrepublik sollten mit einer parallel angestrebten internationalen Anerkennung einhergehen. Die wissenschaftspolitischen Folgen für die Medizin waren der von der SED [Sozialistische Einheitspartei Deutschland] geforderte Austritt der DDR-Ärzte aus westdeutschen Fachgesellschaften, die Gründung von Fachgesellschaften in der DDR mit der Durchführung eigener Kongresse, die Herausgabe von Zeitschriften als Publikationsorgane vorwiegend für DDR Wissenschaftler” (The measures to strengthen the GDR, with increasing demarcation from the Federal Republic, were to go hand in hand with increased international recognition. The scientific–political consequences for medicine were the withdrawal of GDR doctors from West German specialist societies, demanded by the SED [Socialist Unity Party of Germany], the establishment of specialist societies in the GDR with the implementation of their own conventions, and the publication of journals as publication organs primarily for GDR scientists) [79]. The first editor of the *HNO-Praxis* was Kurt Dietzel (1912–2002), Rostock, followed by Hans-Jürgen Gerhard (1928–2010), Berlin, and Lutz Keßler (1936–), Dresden. Kurt Dietzel, who completed his habilitation under Woldemar Tonndorf (1887–1957) in Leipzig in 1954, was full professor for Oto-Rhino-Laryngology in Rostock between 1961 and 1978. Dietzel is credited with the expansion of the journal to include the *Folia broncho-oesophagologica* in 1980, as this signals ENT’s interest in the important endoscopic border areas, in times when endoscopies of the oesophagus and the tracheo-bronchial system were predominantly still carried out with rigid tube endoscopes. In the last issue there was a matter-of-fact “Hinweis an unsere Abonnenten der Zeitschrift HNO-Praxis!” (Note to our subscribers of the journal *HNO-Praxis*) that the journal will be merged with the journal *Laryngo-Rhino-Otologie* from Georg Thieme Verlag Stuttgart in January 1991 [38]. The typical addition “… vereint mit HNO-Praxis …” (… united with HNO-Praxis …), when journals merge, is mentioned in the *LRO* only from 1994 on, which corresponds to the takeover of VEB Georg Thieme Verlag (East), Leipzig, by Georg Thieme Verlag (West), Stuttgart. This coincides with the takeover of the journal *HNO-Praxis* of the Society for Otorhinolaryngology and Cervico-Facial Surgery of the GDR. The GDR ENT doctors, were, however, immediately perceived as subscribers. At the time of the reunification of the two German states, there was great apprehension on the part of the “ENT community” of the Federal Republic about the possible proximity of the GDR professors to the GDR system. It was not until 1993 that a GDR professor was re-elected to the editorial board of the *LRO*, namely Bernd Freigang (1941–), Berlin.

### Title history *ORL: journal for oto-rhino-laryngology and its related specialties*

#### Individual volumes of reports of the scientific spring meeting of the Swiss Society for Otorhinolaryngology, Neck and Facial Surgery, individual volumes of Nederlandse Keel-Neus-Oorheelkundige Vereniging: Vergadering, Basel; Freiburg, Br.; Paris; London; New York, NY; New Delhi; Bangkok; Singapore; Tokyo; Sydney: Karger [19]

In 1908 the journal *Passow-Schäfer Beiträge zur Anatomie, Physiologie, Pathologie und Therapie des Ohres, der Nase und des Halses* was founded in Berlin by the Samuel Karger Verlag (Table 5). The title was issued up to volume 31, in 1934. The founding editors, the otologist Adolf Passow (1859–1926), Berlin, and the physiologist Karl Ludolf Schäfer (1866–1931), Berlin, were able to attract ENT authors from all over the world thanks to their emphasis upon strictly scientific content. They worked together closely since Schäfer became head of the acoustic–physiological laboratory at the Ear Clinic in Berlin in 1907.

**Fig. 11:**
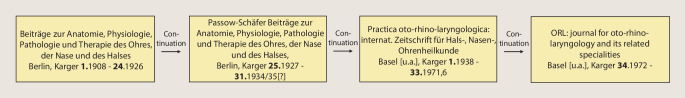
Course of title changes and mergers of the *ORL*

**Fig. 12:**
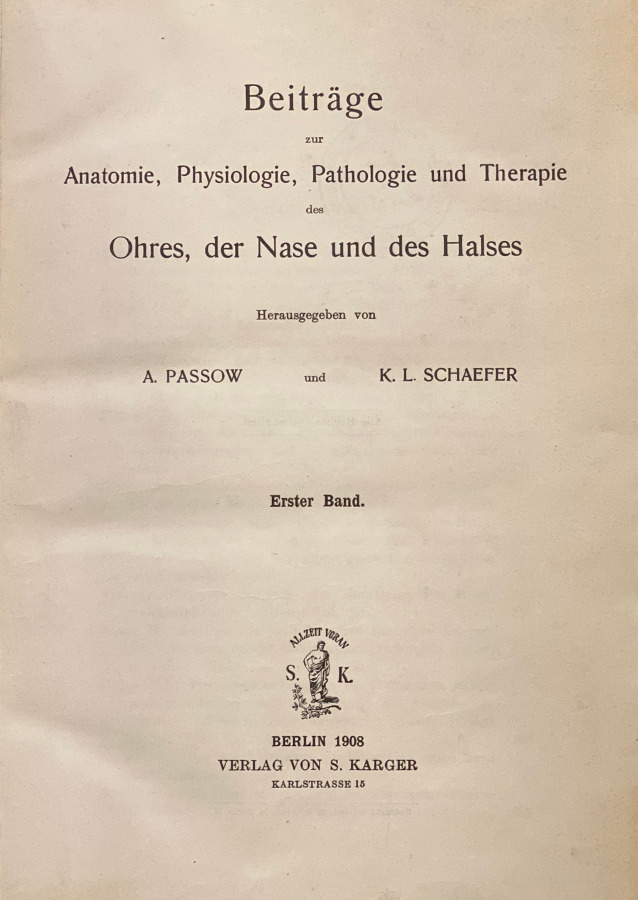
Title page of the first volume of the *Beiträge zur Anatomie, Physiologie, Pathologie und Therapie des Ohres, der Nase und des Halses* from 1908

**Fig. 13:**
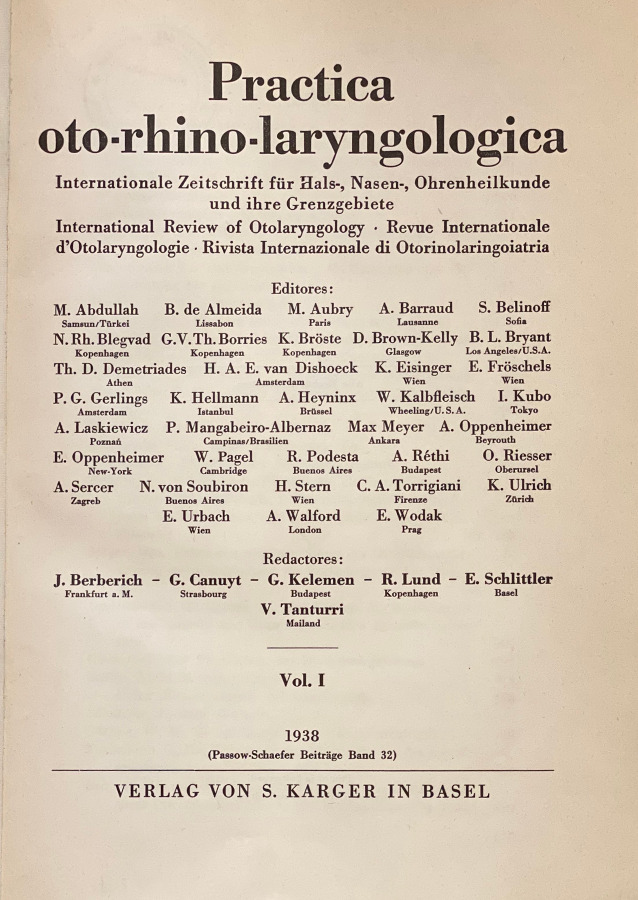
Title page of the first volume of the *Practica oto-rhino-laryngologica* from 1938

When the S. Karger publishing house moved to Basel because of Nazi pressure, the journal was initially discontinued. The journal was relaunched as a new foundation as an international and multilingual journal, *Practica oto-rhino-laryngologica: internat. Zeitschrift für Hals‑, Nasen‑, Ohrenheilkunde, *which was renamed *ORL: journal for oto-rhino-laryngology and its related specialities* in 1972. There was an explicit reference to the predecessor, *Passow-Schäferschen Beiträge*, but the numbering was new. The course of title changes and start-ups is shown in Fig. 11 in a flow chart. The respective title pages of the newly founded predecessor journals of the *ORL* are shown in Figs. 12 and 13.

Due to the bans of the National Socialists, the publishing house lost the majority of its authors and editors for the *Passow-Schäfer Beiträge* in Germany. The journal was discontinued when the publishing house moved to Basel. In the introduction to the opening booklet (volume 1, 1938) of the journal *Practica oto-rhino-laryngologica* published by Karger Verlag, Basel, the publisher Samuel Karger explicitly states that the new journal was founded in the tradition of the *Passow-Schäfer Beiträge zur Anatomie, Physiologie, Pathologie und Therapie des Ohres, der Nase und des Halses* founded 1908 in its own publishing house. This was made visible by adding “Passow-Schäfer-Beiträge” to the journal title [47]. The above-mentioned publication option for other languages was also introduced then [47]. The journal has been the publication organ of the Dutch ENT Society (Nederlandse Keel-Neus-Oorheelkundige Vereniging) since 1963 and was continued from 1972 as the journal *ORL: journal for oto-rhino-laryngology and its related specialties* by S. Karger Verlag, Basel. The *ORL* appears to this day, 2020, as volume 82, and regularly publishes the reports of the scientific spring meeting of the Swiss Society for Otorhinolaryngology, Neck and Facial Surgery [19].

Around 1900, there were also numerous review journals that summarized and reported the increasing number of international publications worldwide for clinicians and practicing physicians. In addition to the editor, numerous otologists, laryngologists and rhinologists worked as translators into German. The review journals lasted for many decades, and were later discontinued with the increasing dominance of English as the language of science. Today this tradition lives on as a column in some current ENT publications, journal clubs in clinics or in commercially organized update events.

### Title history *Internationales Centralblatt für Laryngologie, Rhinologie und verwandte Wissenschaften*

#### Berlin: Hirschwald 1. 1884/85 – 25. 1909, 6, [17]

The oldest German-language review journal including a collection of ENT reports was the *Internationale Centralblatt für Laryngologie, Rhinologie*, founded in 1884. The title was published by Hirschwald Verlag, Leipzig, until 1922. After the takeover by Springer Verlag, the volume numbering was reset and the journal was supplemented by otology. Various name changes were made before discontinuation after volume 148 in 1996. The first editor was the German–British laryngologist Felix Semon (1849–1921). He was able to recruit numerous laryngologists from Europe and North America to collaborate, making publications in most European languages available in German translation. The title history, the renaming and the editors are shown in Table 6. Felix Semon, born in Gdansk in 1849, began his training with the most famous English laryngologist at that time, Morell Mackenzie in London, after studying medicine in Heidelberg and Berlin. After that he quickly advanced his scientific career as secretary of the laryngology subsection of the International Medical Congress [94]. He was later considered one of the most famous laryngologists in the world, lived and worked from then on in London, became a British citizen and was knighted by Queen Victoria in 1897. He ran the *Internationale Centralblatt für Laryngologie, Rhinologie und verwandte Wissenschaften *for a quarter of a century. The title, published in German, was the only international journal for rhinology and laryngology at the time. Up to 2000 papers from up to 15 countries in Europe and North America were discussed annually [28]. Because of his invaluable services to the journal, the publisher August Hirschwald and the new editor Georg Finder decided to rename it *Semon’s Internationales Centralblatt für Laryngologie, Rhinologie und verwandte Wissenschaften* in 1909, after Semon’s retirement as editor. Unfortunately, with the beginning of World War I and the associated spread of nationalistic tendencies in society in 1915, there was a rift between publisher and editor on the one hand and the founder of the journal on the other. After a public statement by Semon in *The Times* on the “… barbarous methods, one and all, employed by Germany” and a reply from Finder and Hirschwald published in the *Centralblatt*, including the decision to rename the journal without Semon’s name, the laryngologist communities of Europe and North America were further polarized. A number of British and American co-editors withdrew from the journal, and various German, Austrian and Hungarian professional societies withdrew their honorary memberships for Semon. The process is described in a commentary in *The Lancet* from 1915 and the name change of the important international journal is criticized as a malicious excess of competence by the publisher Hirschwald (cf. *The Lancet* 1915, pp. 404–405 [4]). The *Internationales Centralblatt für Laryngologie, Rhinologie und verwandte Wissenschaften *came to Springer through the takeover of the Hirschwald publishing house in 1921 and continued to operate as an organ of the scientific ENT society after the World War II, publishing the content of its annual meetings. After various renamings, the publication was discontinued in 1996.

**Table 6:** Title history and editors of *Internationales Centralblatt für Laryngologie, Rhinologie und verwandte Wissenschaften *[17]

1	***Internationales Centralblatt für Laryngologie, Rhinologie und verwandte Wissenschaften***
	Berlin: Hirschwald **1**. 1884/85 – **25**. 1909, 6
	Editors (1884–1909): Felix Semon, London
2	***Semon’s internationales Centralblatt für Laryngologie, Rhinologie und verwandte Wissenschaften***
	Berlin: Hirschwald **25**. 1909, 7 – **31**. 1915, 5
	Editors (1909–1915): Georg Finder, Berlin
3	***Internationales Zentralblatt für Laryngologie, Rhinologie und verwandte Wissenschaften***
	Berlin: Hirschwald **31**. 1915, 6 – **38**. 1922, 3
	Editors (1915–1922): Georg Finder, Berlin
4	***Zentralblatt für Hals‑, Nasen- und Ohrenheilkunde sowie deren Grenzgebiete: Organ der Deutschen Gesellschaft der Hals‑, Nasen‑, Ohrenärzte***
	Berlin: Springer **1**. 1922 – **110**. 1975, 5 (Sept.)
	Editors (1922–1975): Georg Finder, Berlin, Alfred Güttich, Berlin/Cologne, Karl L. Schaefer, Berlin, Hermann Beyer, Berlin/Münster, Artur Blohmke, Frankfurt a.M., Carl v. Eicken, Berlin, Max Meyer, Würzburg, Hermann Frenzel, Göttingen, Werner Kindler, Berlin/Heidelberg, Alfred Seiffert, Heidelberg, Otto Steurer, Hamburg, Woldemar Tonndorf, Dresden/Leipzig, Rudolf Link, Berlin/Hamburg, Otto Novotny, Vienna, Luzius Rüedi, Zurich, Hans-Jürgen Denecke, Heidelberg
5	***Zentralblatt für Hals‑, Nasen- und Ohrenheilkunde, plastische Chirurgie an Kopf und Hals: Organ der Deutschen Gesellschaft für Hals-Nasen-Ohrenheilkunde, Kopf- und Halschirurgie***
	**110**. 1975, 6 (Dec.)(1976); **111**. 1975/76 (1975/77), **1 **(Oct.) – **127**. 1981
	Editors (1975–1981): Rudolf Link, Hamburg, Otto Novotny, Vienna, Luzius Rüedi, Zurich, Hans-Jürgen Denecke, Heidelberg
6	***Zentralblatt Hals-Nasen-Ohrenheilkunde, plastische Chirurgie an Kopf und Hals: Organ d. Deutschen Gesellschaft für Hals-Nasen-Ohrenheilkunde, Kopf- und Halschirurgie*** ***=*** ***Oto-Rhino-Laryngology, plastic surgery of head and neck***
	Berlin; Heidelberg [et al.]: Springer **128**. 1982 – **148**. 1996; thereafter publication discontinued
	Editors (1982–1996): Rudolf Link, Hamburg, Otto Novotny, Vienna, Luzius Rüedi, Zurich, Hans-Jürgen Denecke, Heidelberg

### Title history *Internationales Centralblatt für Ohrenheilkunde*

#### Leipzig: J. A. Barth, 1. 1903 – 5. 1907, [35]

In 1903, the publishing house J. A. Barth, Leipzig, founded a further review journal which was initially devoted exclusively to the processing of international otological literature. Founding editors were Oskar Brieger (1864–1914), Breslau, and Giuseppe Gradenigo (1859–1926), Turin. At that time Brieger was the primary physician at the Allerheiligen Hospital in Breslau. He himself published on otological topics, such as middle ear tuberculosis, the surgical treatment of chronic otitis media and otogenic diseases of the meninges. Gradenigo worked with Politzer in Vienna and in 1889 became director of the University Clinic for Ear and Larynx Patients in Turin. As a co-founder of the *Archivio italiano di Otologia, Rhinologia e Laryngologia* [29], he oversaw the Italian-language publications in the ENT field. Fifteen further otologists from Europe, North and South America were recruited to work on the first volume of the *Centralblatt*. Publications in all important languages at the time, such as English, French, Spanish, Russian and the Scandinavian languages were presented in German translation. As early as 1913, rhinology and laryngology were included. For 22 years, from 1913 to 1935, Franz Kobrak (1879–1955), Berlin, was co-editor of *Internationales Zentralblatt für Ohrenheilkunde und Rhino-Laryngologie*. Born in Breslau, Kobrak completed his specialist training with Brieger in Breslau after studying in Breslau and Munich. In 1907 he settled in Berlin as a specialist. After his habilitation at the Ear Clinic of the Charité near Passow, he was appointed associate professor at the Charité in 1926. He supervised the ear, nose and throat department at the St. Norbert Hospital in Berlin Schöneberg, which was inaugurated in 1914 and destroyed in 1943, and was also extremely active scientifically during this time. As a Jewish doctor and university professor, Kobrak’s teaching license was revoked in 1933 on the basis of Paragraph 3 des Gesetzes zur Wiederherstellung des Berufsbeamtentums vom 7. April 1933 (Sect. 3 of the Law for the Restoration of the Professional Civil Service of April 7, 1933) because of “nicht-arischer Abstammung” (non-Aryan descent). He returned to Berlin after the war. He gave a remarkable lecture on the physiology of labyrinthine fluids at the first post-war congress of the Gesellschaft Deutscher Hals-Nasen-Ohrenärzte (Society of German Ear, Nose and Throat Physicians) in Karlsruhe in 1949. As a result, Kobrak received honorary membership of the German and Austrian Society (cf. Schagen 2013 [74], Jauerneck 1955 [42]). After *Internationales Zentralblatt für Ohrenheilkunde und Rhino-Laryngologie *changed to Curt Kabitzsch publishing house, Leipzig, the title changed to *Der Hals‑, Nasen- und Ohrenarzt Teil 2 Übersichtsberichte u. Referate* in 1936 and then the publication was discontinued in 1943. The title history, name changes and editors are shown in Table 7. Since 1909, the Kabitzsch Verlag had another ENT journal for original papers in its program, the *Zeitschrift für Laryngologie, Rhinologie und ihre Grenzgebiete*, which is still published today as *Laryngo-Rhino-Otologie (LRO)* by Thieme Verlag.

**Table 7:** Title history and editors of *Internationales Centralblatt für Ohrenheilkunde *[35]

1	***Internationales Centralblatt für Ohrenheilkunde***
	Leipzig: J. A. Barth, **1. **1903–**5. **1907
	Editors (1903–1907): Oskar Brieger, Breslau, Giuseppe Gradenigo, Turin
2	***Internationales Zentralblatt für Ohrenheilkunde***
	Leipzig: J. A. Barth, **6. **1908 – **10. **1912
	Editors (1908–1912): Oskar Brieger, Breslau, Giuseppe Gradenigo, Turin
3	***Internationales Zentralblatt für Ohrenheilkunde und Rhino-Laryngologie***
	Leipzig: J. A. Barth, **11. **1913 –** 20. **1922
	Editors (1913–1922): Oskar Brieger, Breslau, Giuseppe Gradenigo, Turin, Max Goerke, Breslau, Bernhard Heine, München, Jörgen Möller, Kopenhagen, Paul Stenger, Königsberg, Franz Kobrak, Berlin
4	***Internationales Zentralblatt für Ohrenheilkunde und Rhino-Laryngologie, Folia oto-laryngologica: Teil 2***
	Leipzig: Curt Kabitzsch, **21**. 1923 – **41. **1935
	Editors (1923–1935): Max Goerke, Breslau, Bernhard Heine, München, Franz Kobrak, Berlin, Jörgen Möller, Kopenhagen, Paul Stenger, Königsberg
5	***Der Hals-Nasen und Ohrenarzt, Teil 2, Übersichtsberichte und Referate***
	Leipzig: Curt Kabitzsch, **42. **1936 – **52. **1943, 1943; thereafter publication discontinued
	Editors (1936–1943): Wilhelm Berger, Königsberg, Alfred Brüggemann, Giessen, Helmut Loebell, Marburg/Münster, Hermann Marx, Würzburg, Jürgen Möller, Kopenhagen, Paul Stenger, Berlin, Alfred Seiffert, Kiel/Heidelberg

### Title history: *Verhandlungen der Gesellschaft Deutscher Hals‑, Nasen- und Ohrenärzte: Jahresversammlung*

#### Berlin; Munich: Bergmann 1. 1921 – 19. 1939, Leipzig: Kabitzsch [initially], and following,

Table 8; [16, 21]

**Table 8:** Titel history and editors of *Verhandlungen der Gesellschaft Deutscher Hals‑, Nasen- und Ohrenärzte: Jahresversammlung*

1	***Verhandlungen der Gesellschaft Deutscher Hals‑, Nasen- und Ohrenärzte: Jahresversammlung***
	Berlin; München: Bergmann **1. **1921 – **19. **1939, Leipzig: Kabitzsch [initially]
	Editors (1921–1931): President and Secretary of the German Society of Ear, Nose and Throat Physicians
2	***Verhandlungen der Deutschen Gesellschaft der Hals‑, Nasen‑, Ohrenärzte: Jahresversammlung***
	Berlin [u. a.]: Springer **20. **1949 – **39. **1968
	Editors (1949–1968): President and Secretary of the German Society of Ear, Nose and Throat Physicians
3	***Verhandlungen der Deutschen Gesellschaft für Hals-Nasen-Ohren-Heilkunde, Kopf- und Hals-Chirurgie: auf der …*** ***Jahresversammlung***
	Berlin; Heidelberg [u. a.]: Springer **40. **1969 – **53. **1982
	Editor (1969–1982): President and Secretary of the German Society of Oto-Rhino-Laryngology, Head and Neck Surgery
4	***Verhandlungsbericht …/Deutsche Gesellschaft für Hals-Nasen-Ohren-Heilkunde, Kopf- und Halschirurgie***
	Stuttgart; New York: Thieme, Berlin; Heidelberg [u. a.]: Springer [initially], 1983–1996,1; 1997,1; 1999–2015, 1996,2; 1997,2 – 1998 not published, irregular, is attachment to: 1993–1996,1 Suppl. to: European Archives of Oto-Rhino-Laryngology, further title remarks: 1990,1–1996,1 at the same time volume of: Deutsche Gesellschaft für Hals-Nasen-Ohren-Heilkunde, Kopf- und Hals-Chirurgie: annual meeting of the Deutsche Gesellschaft für Hals-Nasen-Ohren-Heilkunde, Kopf- und Hals-Chirurgie, single volumes at the same time issue of: Deutsche Gesellschaft für Hals-Nasen-Ohren-Heilkunde, Kopf- und Hals-Chirurgie: Referate, 2001 = **80**, Suppl.1; 2010 = **89**, Suppl. to: Laryngo-Rhino-Otologie, 1983–1992 at the same time all volumes of: [Archives of Oto-Rhino-Laryngology/Supplement]
	Editor (1983–2015): President and Secretary of the German Society of Oto-Rhino-Laryngology, Head and Neck Surgery

**Fig. 14:**
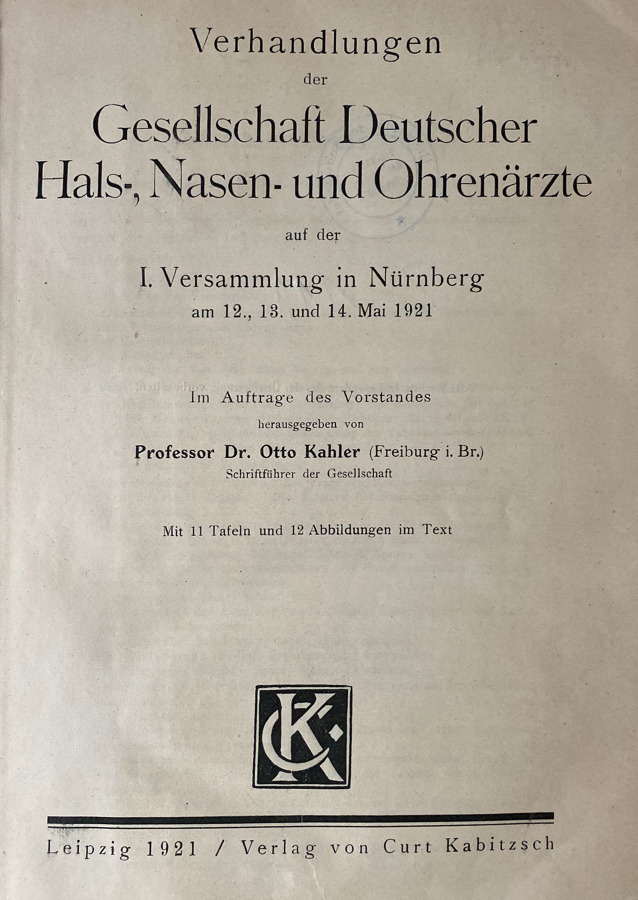
Title page of the first volume of the *Verhandlungen der Gesellschaft Deutscher Hals‑, Nasen- und Ohrenärzte* from 1921

Until 1914 the proceedings of the “Deutsche Otologische Gesellschaft” (German Otological Society) and the “Verein Deutscher Laryngologen” (Association of German Laryngologists) were published in their own journals (by Fischer in Jena from 1895 to 1914 and by Kabitzsch publishing house in Würzburg from 1909 to 1914). With the founding meeting of the “Gesellschaft Deutscher Hals-Nasen-Ohrenärzte” (Society of German Ear, Nose and Throat Physicians) in Nuremberg in 1921, the question of the official publication organ of the new scientific specialist society was discussed. At the first meeting of the new society, chaired by Adolf Passow (1859–1926), Berlin, and Otto Kahler (1878–1946), Freiburg/Br., a committee was commissioned to negotiate with publishers [44]. The *Verhandlungen der Gesellschaft Deutscher Hals‑, Nasen- und Ohrenärzte: Jahresversammlung* was first published by Curt Kabitzsch Verlag in Leipzig and later by Bergmann publishing house in Berlin and Munich. Figure 14 shows the title page of the first volume of the proceedings. The respective presidents and secretaries of the society were the editors of the proceedings. With 92 annual meetings by 2021, listing them is beyond the scope of this publication. They can be found at www.hno.org [15]. Proceedings of annual meetings were subsequently published under different titles in different forms by different publishing houses in print and later in online versions (Tables 8, 9 and 10) and are currently published as a supplement in *LRO* by Thieme Verlag Stuttgart [16]. Presentations and lectures made up the largest part of the publication. Lectures usually represent a summary of current findings, while presentations and posters are like original papers that publish the results of current studies. The form of the presentations has changed over the decades, so that today most of the presentations are only published online in short form as abstracts or as posters. Important discussions during annual meetings were also published until the 1990s. In §21–§29 of the first founding articles of the Gesellschaft Deutscher Hals-Nasen-Ohrenärzte (Society of German Ear, Nose and Throat Physicians), in addition to the duration and order of meetings, the publication of the contributions to the discussion was regulated. Meeting reports and minutes of the Society’s general meetings were initially published in *Verhandlungen*. Since 1976 these have been published in the *HNO-Informationen* by Demeter Verlag, Graefelfing, Balingen and Stuttgart and currently at Deutscher Ärzteverlag, Cologne. The course of the title is shown in Table 8.

**Table 9:** Title history and editors of GMS-CTO (German Medical Science Current topics in otorhinolaryngology—head and neck surgery)

***GMS Current topics in otorhinolaryngology—head and neck surgery: CTO/Deutsche Gesellschaft für Hals‑, Nasen‑, Ohrenheilkunde, Kopf- und Halschirurgie e.*** ***V.***
Düsseldorf: gms proven **3**. 2004-volume **16** (2017); thereafter publication discontinued, https://www.egms.de/dynamic/de/journals/
Editors (2004–2017): Hans-Jürgen Schultz-Coulon, Neuss, Eggert Beleites, Jena, Karl Hörmann, Mannheim, Alexander Berghaus, München, Friedrich Bootz, Bonn, Hans-Wilhelm Pau, Rostock, Gerhard Rettinger, Ulm, Roland Laszig, Freiburg/Br., Norbert Stasche, Kaiserslautern, Heinrich Iro, Erlangen, Thomas Deitmer, Dortmund, Werner Hosemann, Greifswald, Jochen A. Werner, Essen, Dirk Eßer, Erfurt

**Table 10:** Title history of GMS-CPO (GMS Current posters in otorhinolaryngology—head and neck surgery)

***GMS current posters in otorhinolaryngology, head and neck surgery: CPO; …*** ***Jahresversammlung der Deutschen Gesellschaft für Hals-Nasen-Ohren-Heilkunde, Kopf- und Hals-Chirurgie***
Düsseldorf: gms **1**. 2005-volume **13** (2017); thereafter publication discontinued, https://www.egms.de/dynamic/en/journals/
Editors (2004–2017): Hans-Jürgen Schultz-Coulon, Neuss, Eggert Beleites, Jena, Karl Hörmann, Mannheim, Alexander Berghaus, München, Friedrich Bootz, Bonn, Hans-Wilhelm Pau, Rostock, Gerhard Rettinger, Ulm, Roland Laszig, Freiburg/Br., Norbert Stasche, Kaiserslautern, Heinrich Iro, Erlangen, Thomas Deitmer, Dortmund, Werner Hosemann, Greifswald, Jochen A. Werner, Essen, Dirk Eßer, Erfurt

### Title history: *GMS Current topics in otorhinolaryngology—head and neck surgery: CTO*

#### *Deutsche Gesellschaft für Hals‑, Nasen‑, Ohrenheilkunde, Kopf- und Halschirurgie e.V., Düsseldorf : gms Nachgewiesen 3. 2004-volume 16 (2017); (discontinued) and GMS current posters in otorhinolaryngology, head and neck surgery: CPO; … Jahresversammlung der Deutschen Gesellschaft für Hals-Nasen-Ohren-Heilkunde, Kopf- und Hals-Chirurgie,* Düsseldorf: gms 1. 2005-volume 13 (2017); (discontinued), [22, 24]

The board of the German Society for Ear, Nose and Throat Medicine, Head and Neck Surgery decided to publish German-language abstracts and posters, along with English translations in a newly founded electronic journal in 2004. This was primarily for economic reasons and the support of open access publications by the German state. The “GMS—German Medical Science” portal is a product of a cooperation between the Working Group of Scientific Medical Societies (AWMF), the German Institute for Medical Documentation and Information (DIMDI) and the German Central Library for Medicine (ZB MED). According to the Open Access conditions, a creative commons license is used, which guarantees the free availability of the research results obtained through state funding. The electronic journals GMS-CTO and GMS-CPO were available up to volume 16 and 13 respectively and were discontinued in 2017. Since the 89th annual meeting of the German Society for Otorhinolaryngology, Head and Neck Surgery in 2018, the German- and English-language lectures are published in the journal *LRO* Supplement 1, the German- and English-language abstracts and the posters in *LRO* Supplement 2 and are also available online via a link to the website of the scientific society free of charge [16]. Title history and editors are shown in Tables 9 and 10.

## Outstanding publications and paradigm shifts in practical and scientific ENT medicine

Individual publications with a lasting influence on the understanding of diseases, diagnostics or therapy are rare. Such changes in scientific understanding are usually only noticed in retrospect. A historical review provides the ideal framework to identify the achievements of individual scientists and clinicians that lead to paradigm shifts, albeit not without considerable controversy, in ENT. A few will be mentioned here on the basis of publications in German ENT journals. It is not possible, however, to provide an exhaustive review of all important publications and authors of the last 200 years, and the following selection is highly subjective.

In 1873, Hermann Schwartze published “Über die künstliche Eröffnung des Warzenfortsatzes” (On the artificial opening of the mastoid process) in *Archiv für Ohrenheilkunde*. He reactivated a surgical method that had been carried out only sporadically in over a hundred years, and had fallen into disrepute due to incorrect indication and a lack of scientifically founded surgical technique. Schwartze had completed his habilitation in Halle an der Saale in 1863 in the field of ear medicine. At this time severe prejudices caused by earlier errors, weighed on ear medicine (“… schwere Vorurtheile, welche, durch frühere Irrthümer hervorgerufen, auf der Ohrenheilkunde lasteten”) [49]. After years of anatomical, clinical and pathological studies, he and his assistant Adolf Eysell (1846–1934) published “Über die künstliche Eröffnung des Warzenfortsatzes” (On the artificial opening of the mastoid process) in 1873 [83–85]. Eysell was an assistant at the Ear Clinic in Halle an der Saale until 1875 before he practiced as an ear, nose and throat doctor in Kassel starting in 1876. From then on he only published entomological treatises [99]. Schwartze and Eysell were not only the first to define a scientifically based indication for mastoidectomy, but also the surgical technique with hammer and chisel. Schwartze rejected the uncritical, blind trepanation practiced by his predecessors and was able to refute his critics with his published clinical successes. Today, Schwartze is considered to be the father of modern mastoid surgery. Of Schwartze’s first 100 described surgical cases, 20 died and 74 cases were cured. “In keinem Falle war der letale Ausgang mit Bestimmtheit als directe Folge der Operation zu betrachten” (In no case was the fatal outcome to be regarded with certainty as a direct consequence of the operation) [82]. Schwartze described the following indications: subperiosteal abscesses (62 cases), acute inflammation of the mastoid process (17 cases) and pus retention in the middle ear (13 cases). It is also noteworthy that in 9 of 39 cured cases of the second series, a complete normalization of hearing was achieved [82]. These were outstanding and remarkable results at that time, especially considering that the opening of the mastoid process was mostly performed in patients with life-threatening conditions. Mastoidectomy soon became widely accepted. What is interesting about the publication of the evaluation of the first 100 cases is not only the analysis of the individual cases, but also the critical examination of the complications [82]. Despite being retrospective, this publication by Schwartze can be seen as an early form of a clinical study. The *Archiv für Ohrenheilkunde *published mainly case reports, original articles with a predominantly descriptive character, patient statistics from ear clinics, conference proceedings and book reviews in the first few decades [63].

In 1944, a German ENT doctor and prisoner of war in American captivity in Aix en Provence had a lively scientific exchange with American military doctors. His name was Horst Wullstein (1906–1987). He took the opportunity given by Dr. Weiss to study the literature he had brought with him from New York City by Lempert (1891–1968) and Shambaugh (1903–1999) on the results of fenestration surgery, which were becoming increasingly common in the USA at the time [107]. Based on the success of these fenestration procedures in otosclerosis, the idea arose to improve hearing with a similar technique after the surgical repair of chronic otitis of the middle ear. After Horst Wullstein, who was still chief physician in Siegen in the early 1950s, had performed numerous fenestration operations himself, he attempted to apply the principles of sound protection, columella effects and the closure of the eardrum with split-thickness skin grafts to the reconstruction of the middle ear. In almost half of 1000 fenestration operations he performed, he observed poor mastoid pneumatization or direct inflammatory changes in the middle ear. In his original publication “Funktionelle Operationen im Mittelohr mit Hilfe des freien Spaltlappen-Transplantates” (Functional operations in the middle ear with the help of the free split-thickness flap transplant) [105], published in 1952 in *Archiv für Ohren‑, Nasen- und Kehlkopfheilkunde, *he first described the concept of a hearing-improving operation. Wullstein introduced the term “tympanoplasty” and the description of the tympanoplasty types in his contribution to Uffenorde’s surgical textbook [106]. The original work in the *Archiv für Ohren‑, Nasen- und Kehlkopfheilkunde*, however, already described the various surgical techniques in detail, including numerous instructive drawings. The clinical course and audiograms were presented for more than ten patients. In the following years Würzburg became an internationally renowned centre for hearing improvement surgery. The classification of tympanoplasty techniques according to Wullstein is still in common use worldwide.

In 1964, six years after starting his specialist training in ENT under Seiferth in Cologne, Oskar Kleinsasser (1929–2001) first published his concept of “Mikrochirurgie im Kehlkopf” (microsurgery in the larynx) [50]. Kleinsasser, originally trained as a pathologist, had completed his habilitation two years earlier in the early diagnosis of laryngeal cancer. In his original work on microsurgery, he not only described the special problems of adapting a microscope to a suspension laryngoscope, but also developed the technical implementation in cooperation with commercial companies. Over a number of years he had contact with Geza Jako from Boston, MA, connecting him to the development of a loupe laryngoscope into a laryngeal surgical microscope [50]. In addition, it was necessary to develop new laryngeal instruments and laryngoscope tubes. The instruments developed by Kleinsasser for microsurgery in the larynx are still used today and bear his name. A particular problem mentioned in the publication was the insufficient working distance between the binocular surgical microscope and the laryngoscope to insert instruments. A sufficiently large working space was achieved when the microscope manufacturer provided lenses with 400 mm objective focal length [50]. The typical tubular laryngoscopes, long instruments and 400 mm microscope focal length used today go back to the fundamental work of Kleinsasser in the 1960s. These practical developments represented the culmination of a process which had begun some 10 years earlier. Rosemarie Albrecht from Erfurt, inspired by the success of her colleagues in gynaecology in the early detection of malignant lesions on the cervix uteri, had used a borrowed magnifying colposcope for the larynx [1]. The successful collaboration with microscope and instrument manufacturers and the innovative developments by both Geza Jako and Oskar Kleinsasser, led to microlaryngoscopy becoming a globally accepted method for endolaryngeal diagnostics and surgery.

Walter Messerklinger (1920–2001), head of the Graz University ENT Clinic from 1959–1990, is considered the pioneer of endoscopic surgery for chronic sinusitis. His fundamental work “Über die Drainage der menschlichen Nasenebenhöhlen unter normalen und pathologischen Bedingungen” (On the drainage of the human sinuses under normal and pathological conditions) in 1966 [59], he initiated a trend that continues to this day. In the 1980s and 1990s, this led to a change in the philosophy of surgical treatment of chronic rhinosinusitis. Instead of radical mucosa resecting procedures, surgery became increasingly functional using mucosa sparing techniques. The work published in *Monatsschrift Ohrenheilkunde, Laryngorhinologie* not only describes the normal transport of dyed secretion viewed through a surgical microscope in the paranasal sinuses of the human corpse in detail, but also drainage pathways of the diseased sinus mucous membrane during surgery. He was thus able to resolve the dispute about whether it was better to place a maxillary sinus window in the lower or middle nasal meatus. “Bei operierten Kieferhöhlen wird … der neue Ausführungsgang in den unteren Nasengang meist umgangen und das natürliche Ostium benutzt” (A surgically created maxillary sinus window in the lower meatus will be circumvented by the natural cilial transport activity towards the natural ostium) [59]. It took another 20 years until Messerklinger and his student Stammberger (1946–2018) were able to publish the concept of a new pathophysiology of chronic rhinosinusitis [60] and the technique of endoscopic surgery that was based upon it [90, 91]. The articles were published in the mid-1980s in *Laryngologie, Rhinologie, Otologie: Zeitschrift für Hals-Nasen-Ohrenheilkunde*, successor to the *Monatsschrift* and publication organ also of the Österreichische Oto-laryngologische Gesellschaft and Wiener Gesellschaft der Hals‑, Nasen‑, Ohren-Ärzte (Austrian Otolaryngological Society and the Vienna Society of Ear, Nose and Throat Physicians) [18].

Plenary lectures at conferences are rarely about novel or controversial topics, i.e. they usually summarize current knowledge. This makes the key lecture in 1987 all the more remarkable. Wolfgang Steiner (1942–), newly appointed ENT professor and clinic director in Göttingen, received the invitation in 1987 from the President Klaus Terrahe (1935–) for the annual meeting of the German Society for Neck Nose and Throat Medicine, Head and Neck Surgery in Bad Neuenahr. The topic was “Laserchirurgie im HNO-Bereich (Laserchirurgie zur Behandlung von malignen Tumoren des oberen Aerodigestivtraktes)” (Laser surgery in ENT—laser surgery for the treatment of malignant tumours of the upper aerodigestive tract). Steiner had begun to treat malignant tumours of the upper aerodigestive tract with laser surgery in Erlangen in 1979, and was able to report on his experience with 900 patients. The main lecture was published in the supplement volume of the *Archiv für Otorhinolaryngologie* [93]. Steiner himself described the publication as a “persönlichen Erfahrungsbericht” (personal experience report). He described not only the “Möglichkeiten und Grenzen palliativ symptomatischer sowie kurativer Mono- und Kombinationstherapie” (possibilities and limits of palliative, symptomatic and curative mono- and combination therapy), but also formulated for the first time the basics of transoral laser microsurgery for the treatment of malignant tumours of the upper aerodigestive tract. The procedure, indications and contraindications are described in detail for the individual tumour regions and stages. The abandonment of the traditional block resection and defect coverage in advanced tumours represented a break from established concepts. Primary tumour and neck are operated on at different times. Regional lymphatic drainage is treated with an aim to preserve function, and “häufig sogar regional begrenzt” (often even regionally limited). The primary is resected microsurgically, leaving no large defects that require extensive procedures for closure. This paradigm shift in surgical treatment, especially of advanced head and neck tumours, could of course not go unchallenged in 1987. The publication of the main lecture in the *Archivs für Otorhinolaryngologie* also includes the written transcript of the discussion in the lecture hall, a practice which was still customary at the time. In particular, Oskar Kleinsasser, professor in Marburg, strongly criticized the indication for endoscopic resection of advanced vocal fold carcinoma, citing the better overview achieved by an external approach and the possible reconstruction of the resected vocal fold. Kleinsasser also added: “bei Epiglottiskarzinomen und Hypopharynxkarzinomen gehen unsere Meinungen diametral auseinander” (In the case of epiglottic carcinoma and hypopharyngeal carcinoma, our opinions differ diametrically) [93]. He was particularly critical of laser-surgical tumour reduction with subsequent irradiation, and the wound healing sometimes reminded him of an “ausgebrannten Krater” (burned-out crater) [93]. Kleinsasser severely criticized the lack of useful statistics, stating, “from a scientific point of view, the reports only have anecdotal value” (“wissenschaftlich gesehen nur einen anekdotischen Wert”) [93]. The controversial discussions continued at the following annual meetings. Thanks to the persistence and foresight of Wolfgang Steiner, transoral laser microsurgery (TLM) has been globally established as a standard for the treatment of head and neck tumours alongside innovative techniques like transoral robotic surgery (TORS).

## Major medical publishers

### Springer

Julius Springer (1817–1877) is the father of the widely branched Springer family, which, in addition to booksellers and publishers, also produced engineers, lawyers, artists, gallery owners and scientists. The history of the Springer publishing house began in 1832 when Julius Springer, who was born in Berlin, started an apprenticeship at Enslin’s bookstore in Berlin at the age of 15. After his apprenticeship, he spent 4 years traveling as a bookseller, which initially led him to Zurich, where he met Georg Büchner, among others. After 2 years in Switzerland, he moved on and in 1838 he became an assistant to the Stuttgart bookseller Neff, where he first considered founding his own bookstore. After another position in the Parisian bookstore Brockhaus & Avenarius, he returned to Berlin in 1840.

In 1842, the 25-year-old Springer received a license to run a book trade in Berlin, which at that time already had around 100 book sellers. At the time, all of the larger bookstores in Germany had a commission agent in Leipzig who submitted the booksellers’ orders to the publishers and then had the ordered goods delivered. Given the increasing importance of Berlin as a publishing city, it was uneconomical and time-consuming for northern German retailers to have Berlin production come via Leipzig. So Julius Springer became a commission agent. In 1845 he already had 20 foreign contractors and an educational book program, Enslin, making them the most important commissioners in Berlin.

The “Verlag von Julius Springer” initially published mainly political caricatures and writings in the spirit of the Vormärz (the 1848 revolution). His political statements, in which he complained, among other things, of the arbitrary censorship by the Prussian authorities, also led to legal entanglements. Springer was arrested in his bookstore and imprisoned for 8 days for publishing opposition writings.

After the bookstore was sold in 1858, Springer devoted himself exclusively to the publishing business. Gradually, in addition to political writings, he began to focus on specialized literature from the fields of natural sciences and technology. In spring 1871, the founder of the publishing house took his eldest son Ferdinand (1846–1906) into the publishing house and soon after made him a partner. Within a few years, production increased rapidly from 161 titles in 1871 to 200. After the death of the company’s founder Julius Springer in 1877, Ferdinand Springer took his younger brother Fritz (1850–1944) into the publishing house. The publishing house’s first major boom began in the 1880s with the establishment or takeover of numerous journals. These publications, some of which are still published today, set the pace for the development of the company in the following decades and made a decisive contribution to its economic stability. In 1877 the publishing house had four employees, in 1906 there were already 65.

In 1887, the *Therapeutische Monatshefte* was published as Springer’s first medical journal. Among the authors were renowned scientists such as the later Nobel Prize laureate Paul Ehrlich. One year after it was founded, the monthly already had over 4000 subscriptions. Twelve medical publications were published by 1887; 10 years later there were almost 100. Ferdinand and Fritz Springer took their sons Ferdinand the Younger (1881–1965) and Julius the Younger (1880–1968) in the publishing house, who headed it from 1907 on (cf. [73]).

After a prolonged phase of steady growth, there was a temporary decline in 1905 due to an economic depression. Sales declined temporarily by about 20% in all areas, also affecting technology and medicine. As early as 1910, however, annual production exceeded 200 titles for the first time, and another 3 years later the publisher produced 310 titles, 60 of which were journals. In 1914 Springer Verlag was already the second largest publisher in the German Reich with 379 works after the Leipzig Teubner Verlag. The internationalization of science also began at this time; the centre of the scientific world was no longer Germany and Europe, but the USA. English developed into the unchallenged lingua franca of science [86].

Shortly after the outbreak of World War I in August 1914, there was a dramatic drop in sales in the German publishing industry. Fritz Springer stated, “Es wird nichts verkauft” (Nothing is being sold) (cf. Sarkowski 1992, p. 226 ff. [73]). Since sales had fallen so sharply, there was also a lack of funds to finance new projects. Springer Verlag was particularly hard hit by the crisis, as the two heads of the publishing company Ferdinand and Julius Springer had already gone to the front as reserve officers at the beginning of August 1914. In 1915 only 108 titles were produced. The medical publications were particularly hard hit by the decline in production, as many doctors were in the military and those who stayed at home could not continue their literary work due to excessive workloads. The “Handbook of Internal Medicine” is an example of the difficulties experienced at the time. The 186 page chapter on the liver, biliary tract and pancreas was printed at the beginning of the war, the other articles were postponed until after 1918 [73].

In 1918 J. F. Bergmann publishing was taken over. It was more important than Springer publishing in the medical field at the time. Among the titles acquired was the *Zeitschrift für Ohrenheilkunde*, which had emerged in 1878 from the *Archiv für Augen- und Ohrenheilkunde* published by Knapp, Mauthner and Moos. The number of journals published by Springer from 1918 until the end of the inflation more than doubled. This increase was to a large extent due to the acquisition of the publishing houses J. F. Bergmann (1918) with 10 titles and A. Hirschwald (1921) with 12 titles. In addition, there were 18 journals from other publishers as well as 12 newly founded or conversions of previous book series into journals.

The extreme inflation of the post-war period put many German publishing houses in dire straits. Springer was one of the few to expand in these economically difficult times. In 1921, the Hirschwald’sche Buchhandlung, based in Berlin, was taken over along with the *Archiv für Laryngologie und Rhinologie*. When Springer took over, the bookstore only had 12 employees. Within 10 years it increased its workforce to 154 employees, making Hirschwald Germany’s largest academic bookshop under Springer at the end of the Weimar Republic.

In 1931 Springer purchased the Leipzig-based publishing house F. C. W. Vogel, founded in 1730, which proved to be another important expansion. The added journal portfolio was particularly significant, including the *Archiv für Ohrenheilkunde* founded in 1864 and renamed *Archiv für Hals-Nasen- und Ohrenheilkunde* in 1915.

A few weeks after the appointment of Adolf Hitler as Reich Chancellor by Reich President Paul von Hindenburg on January 30, 1933, the “Gesetz zur Wiederherstellung des Berufsbeamtentums” (Law for the Restoration of the Professional Civil Service) brought about a wave of dismissals of non-Aryan and especially Jewish officials and employees. Professors and institute directors also lost their teaching license and the opportunity to work as a journalist in the various publishing houses. Within a short period of time over 2400 scientists were dismissed. The proportion of Jewish authors and editors of book series and journals was relatively high in the natural sciences and thus in the Springer program in particular. Between 1933 and 1938, more than 50 Jewish journal publishers and editors had to retire from Springer Verlag as part of the National Socialist Gleichschaltung of the scientific specialist press (cf. Sarkowski [73]).

Julius Springer the Younger had Jewish grandparents and had to leave the publishing house in 1935 as a result of the “Reichsbürger-Gesetz” (Reich Citizenship Law). In 1941 an ordinance was issued requiring companies with Jewish names to be renamed, so the “Julius Springer Publishing House”, as it had been called since its founding, had to change its name to “Springer Publishing House”. Hirschwald’s Buchhandlung, which was acquired in 1921, also had to be renamed and operated under the name “Lange & Springer” from 1941 on. In 1942 the Reichsschrifttumskammer ordered that after Julius Springer, the Younger Ferdinand Springer also had to leave his company. The sole shareholders were the brothers Tönjes Lange (1889–1961) and Otto Lange (1887–1967), who were managing directors of the publisher’s Vienna branch at the time. Fritz Springer committed suicide in 1944 in order to escape deportation. His brother Ernst Springer, a lawyer in the Reich Debt Administration until 1936, died in 1944 in the Theresienstadt concentration camp (cf. Sarkowski 1992 [73]). In economic terms, the publishing house initially suffered significantly less than during World War I. Domestic sales had risen continuously since the NSDAP (Nationalsozialistischen Deutschen Arbeiterpartei; Nazi Party) came to power, but foreign business collapsed and the increasing Allied air raids on publishing houses and printing plants gradually brought the publishing business to a standstill in 1943.

In the post-war years publishing houses were required to obtain a license from the American occupation authorities. Initially Springer was refused the resumption of various important journals. Later, a number of the publishing house’s major journals were reissued. *HNO* appeared in 1947 and in 1948, the *Zentralblatt für Hals‑, Nasen- und Ohrenheilkunde* was added. In 1949 the *Archiv für Ohren‑, Nasen- und Kehlkopfheilkunde* was published, together with the *Zeitschrift für Hals‑, Nasen- und Ohrenheilkunde*. Reissuing of 59 existing journals and the establishment of another eight between 1946 and 1950 mark the productivity of those years. At the end of 1950 the number of employees in Berlin was 186 and 57 in Heidelberg. In 1958, the company moved from its first headquarters in the west of Berlin to its current location at Heidelberger Platz. Because of the political situation and the isolation of post-war Berlin, Ferdinand Springer the Younger decided to decentralize the publishing business and opened publishing branches in Heidelberg and Göttingen.

In 1948, J. F. Bergmann Verlag, based in Munich and taken over by Springer in 1918, also received a license from the American military government. In 1977 the medical collections of the J. F. Lehmanns publishing house were taken over, and the J. F. Bergmann publishing house moved to its Munich headquarters. In 1989 J. F. Bergmann was integrated into Springer Verlag as part of a further consolidation process.

An internationalization process began in 1964. Springer Verlag founded its first subsidiary outside the German-speaking area in New York. Between 1970 and 1990 further subsidiaries were established in London, Tokyo, Paris, Hong Kong, Barcelona and Budapest. Asia in particular established itself as a market, so in 1978 the translation and publication of Chinese scientific works into English began. The takeover of other publishing houses such as J. F. Lehmanns, Birkhäuser and Steinkopff accelerated Springer’s economic success. In 1988 the publishing house had more than 1000 employees.

As early as 1911, Springer had introduced a collection of medical–biological reports, evaluating the medical literature in Heidelberg. At its peak in 1978, there were 18 different sections with 80 editors and employees. These evaluated 2600 journals from 5600 contributors in 40 countries. A similar project had existed for the field of ENT as the *Zentralblatt für Hals‑, Nasen- und Ohrenheilkunde sowie deren Grenzgebiete* since 1922. Hans-Joachim Denecke (1911–1990), Heidelberg, was responsible for the *Zentralblatt* for many years.

In the 1990s, the publishing industry was in a state of upheaval, particularly due to digitization, which even some traditional companies did not survive economically. Springer also experienced stagnation of growth rates for the first time since the post-war period. The subscription prices for specialist journals had to be increased significantly. Production, shipping and marketing costs increased. In order to meet the new challenges, the online platform “LINK” (later “SpringerLink”) was launched in 1996, offering the opportunity to read and purchase scientific publications online. In 1998 Bertelsmann acquired the Springer publishing house and founded the Bertelsmann Springer publishing group. In 2002 Springer was the world market leader in the field of electronically published publications.

In 2003 Bertelsmann sold the specialist publishing division Bertelsmann Springer to the British private equity companies Cinven and Candover. This was followed by the merger with the Dutch science publisher Kluwer Academic Publishers (KAP), creating the world’s second largest specialist publishing house, “Springer Science+ Business Media”. For Springer, the sale meant extensive restructuring and modernization. In 2006, the Springer publishing group comprised a total of 70 individual publishers with over 5000 employees in 19 countries, and published 1450 journals and around 5000 book titles annually. In 2010, all publications of the publishing house since the founding year 1842 were scanned and made available online. In 2015, the new Springer Nature group emerged from the merger of Springer Science+ Business Media and the majority of Macmillan Science and Education. With 13,000 employees in over 50 countries, the company generates annual sales of around 1.5 billion euros. After Elsevier, Springer is now the world’s second largest publishing house in the fields of science, technology and medicine [86].

The journal *HNO* currently has a circulation of around 1800 copies and an impact factor of 1088 (2019), the highest among the German-language Springer medical journals. The *European Archives of Oto-Rhino-Laryngology* had an impact factor of 1808 in 2018 [87].

### Thieme

After learning the trade of bookseller and then working in the book trade in Leipzig, London, Brussels and Heidelberg for a few years, Georg Thieme (1860–1925) acquired Theodor Fischer publishing house in Kassel and its publishing rights for medical content with the help of his father’s capital. In 1886 he founded his own bookstore in Leipzig. When Thieme registered his specialist publishing company in the commercial register, he was the first German publisher to devote himself exclusively to medical topics. In the early days of the publishing house, the most important source of sales was the *Reichs-Medicinal-Kalender* with a statistical yearbook, collections of laws and new therapeutics, which was distributed until after World War II [89].

In 1887, Thieme took over the *Deutsche medicinische Wochenschrift* (*DMW*), which was founded in 1875 and is still published today. Dr. Samuel Guttmann was a driving force for the publishing house at this time, which profited from pioneering contributions in the field of bacteriology. Emil von Behring published on diphtheria and Shibasaburo Kitasato on tetanus. The greatest influence on the future development of Thieme Verlag and *DMW*, however, was a four-page original article by Robert Koch, which was submitted in November 1890 under the title “Weitere Mitteilungen über ein Heilmittel gegen Tuberculose” (Further communications about a cure for tuberculosis) [53]. The development of tuberculin triggered euphoria in medicine beyond the German Reich and significantly increased the DMW’s reputation and circulation.

Another milestone was the discovery of “X-rays” by the Würzburg physicist Conrad Wilhelm Röntgen, which was published in *DMW* in 1896 along with the famous X‑ray of the hand of Röntgen’s wife [41]. Within 12 months, another 22 original works on this ground-breaking discovery appeared in the *DMW*, giving further impetus to the development of radiation physics, quantum theory and relativity theory. This revolutionized classical physics, and opened the door for completely new diagnostic and therapeutic options in medicine.

The economic development was further fuelled by a sharp increase in the number of medical students and doctors. By 1888, the number of students had tripled to 9000 within a single decade. At the turn of the century, the *DMW* was one of the most important German medical publications alongside the *Berliner klinische Wochenschrift* and *Münchner medizinische Wochenschrift*, even though, at the time, it was almost exclusively focused on internal medicine and bacteriology. Only one surgical opus was found among 78 medical publications by the publishing house in 1900. Only the fields of ophthalmology, gynaecology and ENT were fully developed and differentiated from surgery at that time. Although the *DMW* was open to all specialist disciplines, very little was published by them, and only if the topics were of general medical interest (cf. Staehr 1986 [89]).

The global fame of German medicine at the time contributed to the economic success and expansion of the Thieme publishing house. Numerous translations were made and foreign journal subscriptions established. The portfolio was gradually expanded. Boas/Hesse and Besold publishers were acquired, including the famous anatomy textbook by Rauber and Kopsch. However, there was also fierce competition between new and established publishing houses in the German Reich, with 150 medical journals published in 1890. In order to successfully compete against the big publishing houses like Enke or Springer, Thieme relied on innovative topics and new techniques in addition to reputable authors. Textbooks dealt with new microscopic and endoscopic techniques and were furnished with numerous illustrations. For example, the first edition of “Lehrbuch der Ohrheilkunde” by Louis Jacobson, published in 1893, contained 330 high-quality illustrations. To generate further income, advertisements for medical products were placed in the publisher’s own journals [89].

At the end of the 19th century, only 14 years after it was founded, Thieme Verlag was a flourishing medium-sized company with 92 titles available, including 6 journals. By far the most lucrative publication at that time was the *DMW*, which sold 500,000 copies. More than 10,000 copies of the second largest source of revenue, the *Reichs-Medicinal-Kalender*, were sold annually. These numbers are still quite modest if you compare them with the Berlin book trade company Springer, which at the beginning of the 20th century was already selling more than 100 journal titles with a circulation of around 4 million annually. The publishing house continued to expand until World War I and moved to larger premises again. During the war years, however, books and journals were forcibly managed, so that of the 11 journals only 4 were published in 1918.

In 1919 Bruno Hauff (1884–1963) became a partner in Thieme Verlag, which he took over in 1925 after the death of Georg Thieme [52]. The publishing house has been run as a family business ever since. The post-war years were marked by extreme hyperinflation and economic crises, which also severely affected Thieme Verlag. In November 1923, one US dollar was worth 4 quadrillion German marks. Generating revenue was only possible through the expansion of international business using foreign exchange. This expanded the hitherto predominantly medical publishing program to include scientific fields, which enabled the publishing house to grow further during the economically difficult period of the Weimar Republic.

National Socialism and World War II did not leave Thieme Verlag unaffected. Since Bruno Hauff’s wife was of Jewish descent, the publishing family was directly affected. Hundreds of Jewish publishers and authors were eliminated by purges. The German medical profession offered little resistance to the Nazi appropriation of medicine. As a result, from 1933 onwards, publications by Thieme Verlag became increasingly focused on racial and military medicine. The acts of war themselves brought the publishing activities to a standstill in 1944 when the publishing headquarters in Leipzig were destroyed in an air raid.

Since Leipzig was under Soviet occupation from July 1945 on, the American military administration had moved the publishing house to Wiesbaden. At that time the Allies strictly forbade the printing and distribution of journals and books.

While publishing houses such as Springer or Urban & Schwarzenberg were able to resume their activities at the end of 1945, Thieme Verlag did not receive its license until April 1946. After 19 months of forced interlude due to the war, the first post-war *DMW* was published, and in the same year the publishing house moved to Stuttgart. The components of the publishing house in Leipzig were gradually disappropriated by the Soviet military administration and transferred to VEB Georg Thieme after the GDR was founded. This claimed to be the legal successor to the publisher founded in 1886 and published a number of pre-war journals.

In 1948, an ENT journal was published by Thieme Verlag for the first time under the title *Zeitschrift für Laryngologie, Rhinologie, Otologie und ihre Grenzgebiete* as an organ of the Association of Southwest German Otolaryngologists.

In 1953 Bruno Hauff’s son Günther (1927–2001) became a personally liable partner of the publishing house. In the period that followed, the company continued to grow. Between 1946 and 1963, in addition to 15 journals, around 1100 new or new editions of books were published, 480 of which were licensed abroad in foreign languages. However, there were hardly any publications in specialist areas until the beginning of the 1960s. At that time only three ENT books were kept in the publishing house’s portfolio [89].

Another acquisition was made in 1971 with the takeover of the traditional Enke publishing house, which is now a leader in the field of specialist veterinary literature. The corporate design of Thieme Verlag with the blue–blue–white tricolor, which is still in use today, was created in 1972. In 1979 Thieme intensified its international activities and founded a subsidiary in the USA, Thieme Stratton Inc., now Thieme Publishers. In 1980 the Hippokrates Verlag was integrated. In addition to *Rhinologie, Laryngologie, Otologie*, three other ENT journals were published by ThiemeVerlag: *Sprache-Stimme-Gehör* since 1977, *The American Journal of Otology* since 1979 and *Seminars in Speech, Language and Hearing* since 1980 (*Hearing* has been an independent journal since 1983). In 1982 Albrecht Hauff joined the publishing house and has been managing the company as a personally liable partner since 1990. In the 1980s and 1990s, various other companies and publishing programs were acquired, in particular the Sonntag Verlag, Parey Verlag, Karl Demeter Verlag and Karl F. Haug Verlag. The TRIAS Verlag was founded for the patient and lay market.

In the course of German reunification in 1990, the VEB Georg Thieme Verlag Leipzig was transferred back from the Treuhandanstalt to Thieme Stuttgart for the symbolic price of DM 1. The former head office in Leipzig was closed in 1992 [55]. The first electronic books were published by Thieme in 1998. Today the entire range of textbooks and many monographs are available in the Thieme E‑Book Library as e‑books. With eRef, a comprehensive medical knowledge platform went online in 2015 [32].

In the course of further publishing acquisitions and content-related development strategies, Thieme Verlag changed into a publishing group. International activities currently account for around 25% of sales. At the turn of the millennium, annual revenues of around 100 million euros had been generated worldwide. The various companies and programs of the publishing group were merged in 2002 within the newly founded company subsidiary Medizinverlage Stuttgart. The number of employees continued to rise from around 600 in 1990 to currently around 1000 with annual revenues of 162 million euros (2018) [12]. *Laryngo-Rhino-Otologie* is currently published in a monthly print run of 1900 copies (impact factor 2019: 0.972), *Sprache-Stimme-Gehör* has a monthly print run of 3100 copies (impact factor 0.3).

### Karger

Siegbert Samuel Karger (1863–1935) founded the “Verlag von S. Karger” in Berlin in 1890 at the age of 27, after completing his training as a bookseller in Posen (now Poznan, Poland) and then working as a bookseller for a few years, most recently in the Berlin bookstore Stuhr. Karger’s primary goal was to present the entire field of medicine through a collection of various compendia. The *Obstetric Vademecum* was published as the first compendium in the year the company was founded [45]. The first periodical was the *Dermatologische Zeitschrift* in 1893 which is still published today. In 1908 Karger published the first ENT journal under the title *Passow-Schaefer-Beiträge zur Anatomie, Physiologie, Pathologie und Therapie des Ohres, der Nase und des Halses* [69].

At the beginning of World War I, Karger published 9 journals and in 1915 alone, around 50 new books. The economic crisis of the following years also left its mark on the Karger publishing house. Karger’s son Heinz (1895–1959) joined the publishing house after completing his business studies at the beginning of the 1920s and took it over after the death of his father in 1935. Until then, the company had grown again after economically difficult years and was already listing more than 850 titles [46, 75].

Like many other publishers of the time, Samuel Karger and his descendants were Jews. Under pressure from the Gestapo, numerous Jewish scientists had to leave the group of authors and editors. In order to avoid the increasing political reprisals, the publishing house relocated to Switzerland in 1937 with the support of the Basel Medical Faculty. By changing to a multilingual concept, the journal was able to access international markets and gain new subscriptions despite the difficult political and economic situation. The titles of the journals were changed to Latin. The *Archiv für Verdauungskrankheiten *was called *Gastroenterologia* and the *Passow-Schaefer-Beiträge* were published from 1938 under the title *Practica Oto-Rhino-Laryngologica*. Articles were published in German, French, English and Italian with summaries in the other languages.

Due to its neutrality, Switzerland was spared active acts of war during World War II, but it was almost completely cut off from the rest of the world. The publisher was now barely able to reliably supply its customers with publications and get manuscripts to Basel, which meant that a large portion of its income was lost. Thanks to the mediation of the Swiss biochemist Guggenheim, funds from various Swiss pharmaceutical companies (e.g. Hoffmann-La Roche, Ciba, Sandoz) were used to secure the existence of the publishing house, which was in a critical economic state [56]. During the war, the publisher stockpiled and was able to quickly sell these stored stocks in the post-war period. Heinz Karger died in 1959 and his son Thomas (1930–2020) took over the management of the publishing house at the age of 29. In contrast to many other publishers, Karger did without in-house specialist editors and instead opted for a purely expert procedure at an early stage.

In the process of internationalization, a worldwide network of publishing agencies was established and English was introduced as the leading language. In 1992 Karger founded a German publishing location in Freiburg im Breisgau, which is primarily aimed at specialists in German-speaking countries. In 1995 around 10% of the submitted manuscript pages in the entire publishing house were still published in German [48]. Gabriella Karger (1964–) has been chairwoman of the company’s board of directors since 2018. S. Karger AG currently has around 240 employees and publishes around 100 medical and scientific journal titles annually, and around 50 book titles (annual sales of $ 280 million) [67]. The *ORL-Journal for Oto-Rhino-Laryngology, Head and Neck Surgery* has an impact factor of 1.012. Between 1991 and 2003 another ENT journal, the *Oto-Rhino-Laryngologia nova: europäische Zeitschrift für Praxis, Klinik und Forschung*, was published by Karger Verlag.

### Urban and Schwarzenberg

Ernst Urban (1838–1923) and Eugen Schwarzenberg (1838–1908) founded a travel and mail order bookstore in Vienna in 1866. With the takeover of the *Wiener Medizinischen Presse* in 1876, the foundation for the medical and scientific focus of the publishing house was laid. In 1879 the *Deutsche Ärztekalender*, which is still published today, was founded. In 1898 Eduard Urban (1875–1953), the 22-year-old son of the company founder, founded a branch in Berlin. His twin brother Karl (1875–1930) later took over the company headquarters in Vienna. In 1901 the Berlin specialist bookstore Oscar Rothacker was taken over. In 1904 the *Medizinische Klinik*, which is still issued today, appeared. In 1909 the publishing house took over the *Monatsschrift für Ohrenheilkunde und Laryngo-Rhinologie*, founded in 1867 and published by the commission publisher O. Coblentz. From 1910 this appeared as an organ of the Austrian otological, the Vienna laryngological and the Munich laryngo-otological societies in an expanded form. The period of World War I and the subsequent decade were years of severe economic recession, inflation, expansion and renewed economic crisis. In 1920 the Berlin laryngologist Gustav Killian published a description of suspension laryngoscopy which significantly influenced laryngology. The 77-page book contained 44 illustrations. During this time, the *Handbuch der biologischen Arbeitsmethoden* (Handbook of biological working methods), which appeared from 1925, was also the most extensive work in the history of the publishing house, comprising 107 volumes.

After the National Socialists came to power in 1933, the Jewish employees at Urban & Schwarzenberg had to leave the publishing house. The works of the Jewish authors could still appear in the Vienna House until the “annexation” of Austria in 1938. In 1943 the Berlin publishing house was destroyed in a bomb attack.

Immediately after World War II, Eduard Urban’s son Heinz (1905–1979) acquired the medical book program from J. F. Lehmanns publishing house, including the famous Sobotta *Atlas der Anatomie des Menschen* (Atlas of Human Anatomy).

In 1946, Eduard Urban received a publishing license from the American military administration and founded a branch in Munich, which in 1949 became the company’s headquarters. The Viennese publishing branch could only be continued as a GmbH by the previous shareholders after 1954. In 1966, on the 100th birthday of the publishing house, the great-grandson of the company founder, Michael Urban, born in 1939, became a partner [95]. In 1976 another branch was founded in Baltimore, MD, USA. Rothacker has meanwhile developed into one of the leading mail-order bookstores for physicians. In 1984 the *Roche Lexikon Medizin* was published with the support of the Hoffmann-La-Roche company [97].

In 1998 Urban & Schwarzenberg was taken over by the Georg von Holtzbrinck publishing group, and a little later merged with the publishing house Gustav Fischer, also belonging to Holtzbrinck. The company was then called Urban & Fischer. In 1999 Urban & Fischer took over the Ullstein Medical Verlag program, making Urban & Fischer the second largest medical book publisher in German-speaking countries. In 2003 Holtzbrinck sold Urban & Fischer Verlag to the Dutch science publisher Elsevier. Elsevier employed 7900 persons in 2018 and published 470,000 peer-reviewed articles in the same year, which corresponded to 18% of the global scientific output [27]. Elsevier has come under global criticism from numerous scientific institutions because of its pricing and refusal to provide open access data.

### Stahel

Johann Jakob Stahel (1723–1787) founded the Stahel’sche royal Bavarian court and university book and art store in Würzburg in 1753. In 1763, with the consent of the royal government, he acquired Kleyer’s university book printing company in Würzburg. For this he had to undergo an apprenticeship as a printer at the age of 40, after which he was finally awarded the title of “Hochfürstlicher Hofbuchhändler” [64]. In 1882 the Stahel’sche Buchhandlung was awarded the title of university bookshop by the academic senate of the University of Würzburg, after the publisher “participated in the secular celebration of the Alma Julia Maximilianea in such an excellent way by publishing various commemorations” (“durch Herausgabe verschiedener Festschriften an der Säcularfeier der Alma Julia Maximilianea in so hervorragender Weise betheiligte”) [64]. The main focus of the publishing program was originally Catholic theology, but in the middle of the 19th century it turned to medical, scientific and legal fields. Well-known publications included *Cannstatt’s Jahresbericht der Medicin* (1851–1865), *Cannstatt’s Jahresbericht der Pharmacie* (1851–1865) and the *Archiv für Ohrenheilkunde* (1864–1873) edited by Anton von Tröltsch, Adam Politzer and Hermann Schwartze [14, 77]. In the period that followed, Stahel no longer published any ENT periodicals. The traditional bookstore was closed on February 15, 1992 for economic reasons.

### Hirschwald

August Hirschwald (1774–1848) founded a bookshop in Berlin in 1816. He started publishing in 1826 and his publishing house developed into one of the most important medical publishers in the German Reich. In 1840 the publishing house and sales were separated, the latter continued under the Hirschwald’sche Buchhandlung company. Specialization in medicine and natural sciences took place very early. After August Hirschwald’s death, his son Ferdinand Hirschwald (1826–1899) joined the company as a partner in 1848, who now ran the publishing house together with Hirschwald’s nephew Eduard Aber (1810–1899) and managed it during its heyday. Important authors included Virchow, Langenbeck, Billroth, Esmarch and Helmholtz. The *Berliner medicinische Central-Zeitung* (1832), the *Berliner klinische Wochenschrift* (1864) and the *Archive für klinische Chirurgie, Gynäkologie oder Laryngologie *(since 1893) [76] were important journals founded at the time. Eduard Aber’s son Albert (1842–1920) continued the company, but was unable to maintain the publishing house’s economic position due to increasing competition. During World War I, production almost came to a standstill and after Aber’s death in 1920 there was no successor within the family. In March 1921 the publishing house and bookstore were taken over by Springer for 175,000 Marks (cf. Sarkowski 1992, pp. 245 f [73]).

### Vogel

In 1808 Friedrich Christian Wilhelm Vogel (1776–1842) acquired a bookstore from L. Crusius (1738–1824) after training as a bookseller in Leipzig. The bookstore traced back to a business founded by Teubner (1695–1757) in Braunschweig in 1730, which was moved to Leipzig and transformed into a scientifically oriented publishing business. Crusius took over from 1764 on and finally handed it over to his colleague F. C.  W. Vogel, who ran it until 1836. In addition to a printing company, Vogel founded a consignment and assortment business. In 1847, Wilhelm Vogel (1808–1872), a son of F. C. W. Vogel, bought the Göttingen university bookstore from Johann Christian Dieterich. In 1862 Carl Lampe-Vischer (1836–1907) took over the publishing business while retaining the old company.

Due to his interest in medicine, the publishing program was increasingly filled with works from this area. One of the best-known otological contributors was the ophthalmologist Anton von Tröltsch (1829–1890). In 1862 Vogel published his *Textbook of Ear Medicine*. The journal *Archiv für Ohrenheilkunde*, which he founded together with Adam Politzer and Hermann Schwartze in 1864 and initially published by Stahel, was also published by Vogel in Leipzig from 1873 onwards. In 1890 the son Carl Friedrich Lampe-Vischer (1864–1937) joined the company and took it over in 1907 after the death of his father. The complete catalogue of Vogel in 1930 showed almost 1000 titles, of which around 500 are still available [39]. Since the beginning of World War I, however, there have only been around 100 new publications. Lampe-Vischer sold the publishing house to Springer in 1931 because his sons were not interested in continuing the business. Up until 1940 the journals of the publisher F. C. W. Vogel were still published by Springer under the old publisher name, including the *Archiv für Ohren‑, Nasen- und Kehlkopfheilkunde* founded in 1864 (cf. Sarkowski 1992, p. 311 f. [36, 73]).

### Barth

Johann Philipp Haug (1747–1784), born in Strasbourg, founded a bookselling business in Leipzig in 1780 without a specific publishing spectrum. In 1784 his widow Catharina Wilhelmina Haug (1755–1799) took over the bookstore. Johann Ambrosius Barth (1760–1813) married C. W. Haug, who assigned the company to him. Scientific works like the *Annalen der Physik* were increasingly included in the publishing program from 1809 on. When he died of an infectious disease while helping in a military hospital in 1813, his son Wilhelm Ambrosius Barth (1790–1851) took over the publishing house at the age of 23 and expanded the scientific spectrum. However, he committed suicide due to financial difficulties, whereupon his son Adolph Ambrosius Barth (1827–1869) took over the bookstore in 1852. Adolph Ambrosius Barth died of typhoid in 1869, leaving behind a well-structured and prosperous company. His brother Johann Ambrosius Barth (1834–1887) then headed the company. After his death, the publishing house was sold to Arthur Meiner (1865–1952), the son of a wealthy Leipzig businessman, in 1890 [88].

Meiner led the company until his death in 1952. He expanded the spectrum through the purchase of journals from Breitkopf & Härtel and the acquisition of the publishing houses Ambrosius Abel, Leipzig, Quandt & Handel, Leipzig, Curt Kabitzsch, Würzburg, Leopold Voss, Hamburg and Hermann Meusser, Berlin. Famous contributing authors included Arrhenius, Boltzmann, Einstein, Helmholtz, Hertz, Planck, Röntgen and Schrödinger. From 1903 to 1921, J. A. Barth published the *Internationale Zentralblatt für Ohrenheilkunde*, edited by Brieger and Gradenigo [43].

Meiner was active in a variety of ways. He was as a board member of the Börsenverein des Deutschen Buchhandels in Leipzig (1918–1923), city councillor (1907–1915), commercial judge (1909–1915) as well as in the Gewandhaus directorate and received an honorary doctorate from the University of Giessen in 1918. In 1943 the publishing house in Leipzig was badly damaged in a bomb attack and the publishing activities came to a standstill in the course of 1944.

At the end of 1945 the publishing house received licenses from the Soviet military administration to continue its activities. In 1947, a further J. A. Barth Verlag was founded in Munich to secure the future of the publishing house. In 1969 it went to the American publisher “Academic Press” and in 1974 to Springer.

After Arthur Meiner’s death in 1952, his widow Hertha Meiner (1875–1964) took over the business. She died in 1964. The daughter and heiress, Annemarie Meiner (1895–1985), had no access to the Leipziger Barth Verlag from Munich because the limited partner shares of the brothers Helmut Meiner (1901–1980) and Wolfgang Meiner (1897–1977), who lived in the Federal Republic of Germany, were administered in trust by the State Bank of the GDR. In 1966, Annemarie Meiner authorized the previously appointed Leipzig managing director, Klaus Wiecke, to continue managing the publishing house. After the death of all three Meiner heirs, VEB Johann Ambrosius Barth Verlag für Medizin, Stomatologie und Naturwissenschaften, (Publishing House for Medicine, Stomatology and Natural Sciences), Leipzig, was sold to the Gruppe VE Verlage für Medizin und Biologie (Publishing Group for Medicine and Biology) Berlin, Jena, Leipzig and transferred to public ownership by the State Bank and the City of Leipzig. In 1991 the company was finally sold by the trust company to the Hüthig Group, and in 1999 the Barth shares went to the Thieme Group. In the same year, the branch office in Leipzig was closed (cf. Links 2009, pp. 102–105 [55], cf. Wiecke 1980, pp. 70 ff [102]).

### Bergmann

Joseph Friedrich Bergmann (1849–1917) founded his publishing house in Wiesbaden in 1878, taking over the medical division of Christian Wilhelm Kreidel (1817–1890) with 78 titles early on, including the *Archiv für Augen- und Ohrenheilkunde* published by Knapp, Mauthner and Moos, which he soon divided into the *Archiv für Augenheilkunde *and *Zeitschrift für Ohrenheilkunde*. After Kreidel’s death in 1890, the entire publishing house founded in 1843, including the fields of science and technology, went to Bergmann, who secured the services of his nephew Wilhelm Geck. After Bergmann left the company due to illness in 1914, Geck continued to run the publishing house [7, 72, 78].

Bergmann made an early agreement with his friend Fritz Springer that Springer Verlag should take over his shares in the publishing house after his death. In 1929 Geck’s shares also went to Springer. Bergmann had an extensive medical program that was more important than that of Springer Verlag at the time of the takeover. Bergmann’s particular strengths lay in the subjects of ophthalmology and ear, nose and throat medicine, which were hardly represented at Springer.

Since Wiesbaden was occupied by French troops up to 1930 after World War I, exchange with authors, printers and bookshops was difficult, which is why the Bergmann publishing house was moved to Munich in 1920. From here Bergmann developed further as a publishing house, with around 300 new publications by 1928. Since then, Bergmann Verlag in Munich has continued to operate with a small staff and mainly looks after its journals and the new editions of older titles. Delivery was moved to Berlin in 1930. In 1991 Bergmann had 166 employees under the umbrella of Springer in Munich (cf. Sarkowski 1992, p. 234 [73]).

## Declarations

For this article no studies with human participants or animals were performed by any of the authors. All studies performed were in accordance with the ethical standards indicated in each case.

The supplement containing this article is not sponsored by industry.
